# Identification of host transcriptome-guided repurposable drugs for SARS-CoV-1 infections and their validation with SARS-CoV-2 infections by using the integrated bioinformatics approaches

**DOI:** 10.1371/journal.pone.0266124

**Published:** 2022-04-07

**Authors:** Fee Faysal Ahmed, Md. Selim Reza, Md. Shahin Sarker, Md. Samiul Islam, Md. Parvez Mosharaf, Sohel Hasan, Md. Nurul Haque Mollah

**Affiliations:** 1 Department of Mathematics, Jashore University of Science and Technology, Jashore, Bangladesh; 2 Bioinformatics Lab., Department of Statistics, Rajshahi University, Rajshahi, Bangladesh; 3 Department of Pharmacy, Jashore University of Science and Technology, Jashore, Bangladesh; 4 Department of Plant Pathology, Huazhong Agricultural University, Wuhan, Hubei Province, China; 5 Department of Biochemistry and Molecular Biology, Rajshahi University, Rajshhi, Bangladesh; Government College University Faisalabad, PAKISTAN

## Abstract

Severe Acute Respiratory Syndrome Coronavirus-2 (SARS-CoV-2) is one of the most severe global pandemic due to its high pathogenicity and death rate starting from the end of 2019. Though there are some vaccines available against SAER-CoV-2 infections, we are worried about their effectiveness, due to its unstable sequence patterns. Therefore, beside vaccines, globally effective supporting drugs are also required for the treatment against SARS-CoV-2 infection. To explore commonly effective repurposable drugs for the treatment against different variants of coronavirus infections, in this article, an attempt was made to explore host genomic biomarkers guided repurposable drugs for SARS-CoV-1 infections and their validation with SARS-CoV-2 infections by using the integrated bioinformatics approaches. At first, we identified 138 differentially expressed genes (DEGs) between SARS-CoV-1 infected and control samples by analyzing high throughput gene-expression profiles to select drug target key receptors. Then we identified top-ranked 11 key DEGs (SMAD4, GSK3B, SIRT1, ATM, RIPK1, PRKACB, MED17, CCT2, BIRC3, ETS1 and TXN) as hub genes (HubGs) by protein-protein interaction (PPI) network analysis of DEGs highlighting their functions, pathways, regulators and linkage with other disease risks that may influence SARS-CoV-1 infections. The DEGs-set enrichment analysis significantly detected some crucial biological processes (immune response, regulation of angiogenesis, apoptotic process, cytokine production and programmed cell death, response to hypoxia and oxidative stress), molecular functions (transcription factor binding and oxidoreductase activity) and pathways (transcriptional mis-regulation in cancer, pathways in cancer, chemokine signaling pathway) that are associated with SARS-CoV-1 infections as well as SARS-CoV-2 infections by involving HubGs. The gene regulatory network (GRN) analysis detected some transcription factors (FOXC1, GATA2, YY1, FOXL1, TP53 and SRF) and micro-RNAs (hsa-mir-92a-3p, hsa-mir-155-5p, hsa-mir-106b-5p, hsa-mir-34a-5p and hsa-mir-19b-3p) as the key transcriptional and post- transcriptional regulators of HubGs, respectively. We also detected some chemicals (Valproic Acid, Cyclosporine, Copper Sulfate and arsenic trioxide) that may regulates HubGs. The disease-HubGs interaction analysis showed that our predicted HubGs are also associated with several other diseases including different types of lung diseases. Then we considered 11 HubGs mediated proteins and their regulatory 6 key TFs proteins as the drug target proteins (receptors) and performed their docking analysis with the SARS-CoV-2 3CL protease-guided top listed 90 anti-viral drugs out of 3410. We found Rapamycin, Tacrolimus, Torin-2, Radotinib, Danoprevir, Ivermectin and Daclatasvir as the top-ranked 7 candidate-drugs with respect to our proposed target proteins for the treatment against SARS-CoV-1 infections. Then, we validated these 7 candidate-drugs against the already published top-ranked 11 target proteins associated with SARS-CoV-2 infections by molecular docking simulation and found their significant binding affinity scores with our proposed candidate-drugs. Finally, we validated all of our findings by the literature review. Therefore, the proposed candidate-drugs might play a vital role for the treatment against different variants of SARS-CoV-2 infections with comorbidities, since the proposed HubGs are also associated with several comorbidities.

## 1 Introduction

The severe acute respiratory syndrome coronavirus (SARS-CoV) is an alarming global health concern starting from the early 21^st^ century. Now this virus is known as SARS-CoV-1. The SARS-CoV-1 is a feverish respiratory tract disease which was first identified in Guangdong Province, China in 2002. It then spread to 29 countries and was first officially recognized in March 2003 [1]. This virus is named Coronaviruses (CoVs) because of its characteristic halo structure under an electron microscope (corona, crown-like). Latin word “corona” means crown or “halo” and coronavirus particles display a crown-like fringe typically referred to as “spikes” under electron microscopy. The CoVs are non-segmented single-stranded RNA viruses covered with envelop which can cause illness ranging in severity from the common cold to severe and fatal illness or even death. On the basis of serotype and genome, the coronavirus subfamily is divided into four genera: α, β, γ and which has long been recognized as important veterinary pathogens that causes severe to lethal respiratory and enteric diseases in birds as well as mammals. More than 8,000 cases of infection and 774 deaths were reported worldwide due to the outbreak of this coronavirus (CoV) between March 2003 and July 2003 [2]. During the outbreak, the average mortality rate was around 9.6% [3, 4]. Koch’s postulated that SARS-CoV-1 was related to pathogenesis and poses a significant threat to human health [5]. Acute respiratory distress syndrome (ARDS) was developed in 16% of the total SARS-CoV-1 patients and the mortality rate became 50% of these types of SARS-CoV-1 patients [6, 7].

Consequently, the COVID-19 was officially declared a pandemic by the WHO on 11 March. Currently, the COVID-19 pandemic is a new global health concern worldwide forcing to adopt lockdown strategies and putting the world health care system in serious crisis with the economic instability. As of 30 July, 2021, around 4.2 million peoples died out of 197 million SARS-CoV-2 infections and gradually infected peoples are increasing worldwide. The infection and death rate were increased exponentially. Current studies have shown that SARS-CoV-2 has a genomic structure close to that of other beta-coronavirus [8, 9]. Recent studies concentrated on genes with SARS-CoV-2 sharing almost 80% nucleotide identity and 89.10% nucleotide similarity with SARS-CoV-1 genes [10, 11]. SARS-CoV-2 is the seventh known human coronavirus (HCoV) from the same family after the pandemic of 229E, NL63, OC43, HKU1, MERS-CoV and SARS-CoV-1 [12]. Because, SARS-CoV-1 and SARS-CoV2 has the great characteristics similarities with respect to its genomic and structural configuration basis on their genetic homogeneity and proximity confirmed by homology alignment of these genes sequence, focusing on receptor binding domain, host cell entry and protease activation. Both of these virus use ACE2 (angiotensin-converting-enzyme2) as entry receptor and human protease as entry activators [13–15]. The spike protein (S) of SARS-CoV-2 interact with ACE2, the same receptor used by SARS-CoV-1 and CoVNL63 to enter the host cells in particular alveolar epithelial cells [16, 17]. This findings surely gives strong evidence that SARS-CoV-1 and SARS-CoV-2 has a link of interaction and might be similar in action in case of infection and disease. Therefore, therapeutic drugs for SARS-CoV-1 infection might be useful for SARS-CoV-2 infections also.

**Fig 1:**
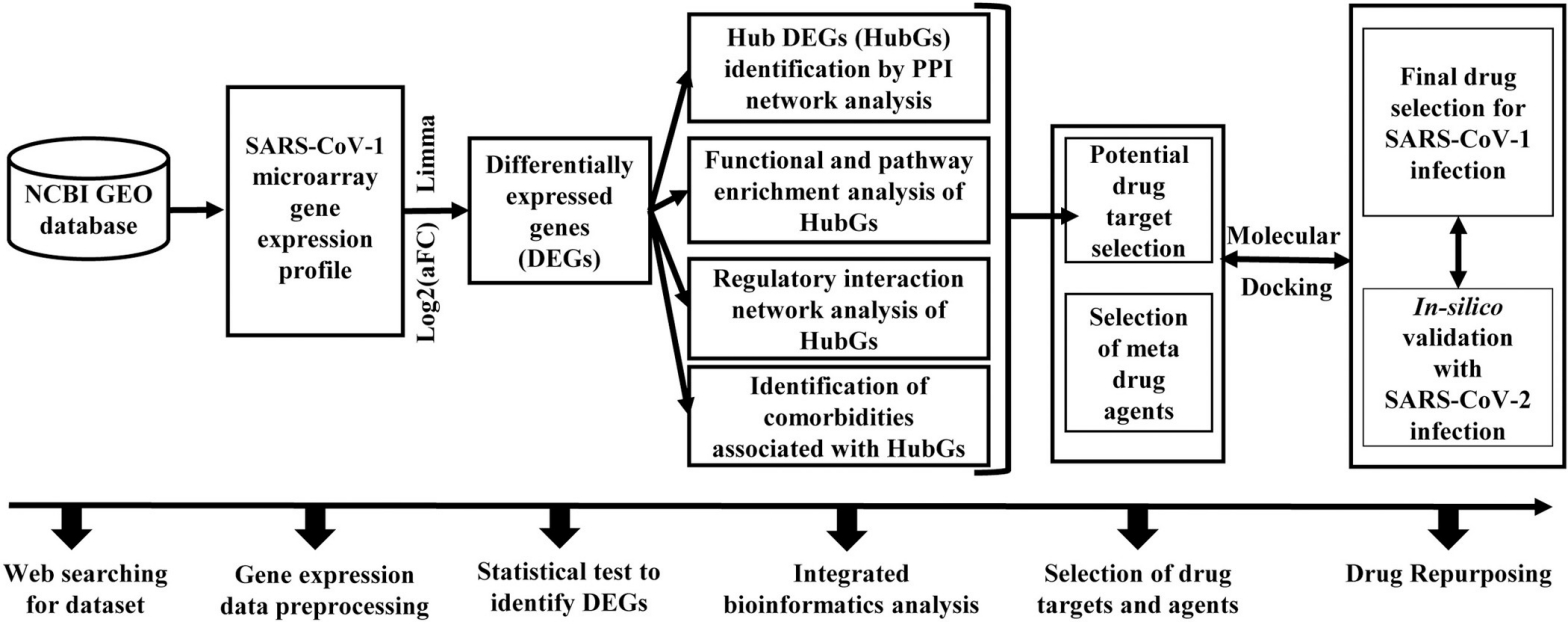
The overview of this study.

Though a number of vaccines including Pfizer, Moderna, Sputnik, AstraZeneca, Ad5-nCoV, EpiVacCorona, BBIBP-CorV, BBV152, CoronaVac, and WIBP are now available against SARS-CoV-2 infections and some are in progress [18, 19], we are worried about their effectiveness due to unstable patterns of coronaviruses. For example, recently we observed that some already vaccinated people got infected by SARS-CoV-2 in our surrounding. Therefore, besides the vaccines, different variants of supporting drugs are also required for the treatment against coronavirus. However, de-novo (new) drug discovery is a tremendous challenging, time consuming and expensive task due to several steps involved in this process from the target based drug selection to the clinical validation. Drug repurposing (DR) is a promising approach to overcome many of those obstacles in discovering and developing new drugs by exploring the new therapeutic applications of FDA approved known drugs that were established for different diseases. It is considered as the supporting process to the conventional drug discovery. To explore more suitable repurposable drugs for a new disease, it requires identifying appropriate target proteins (biomolecules) associated with the new disease. Both host and viral genomic biomarkers mediated proteins (disease related) are considered as the key drug target proteins [20–24]. Beck et al. [25] proposed a list of SARS-CoV-2 SARS-CoV-2 3CL protease-guided repurposable drugs for the treatment against SARS-CoV-2 infections by molecular docking analysis between few SARS-CoV-2-Spike proteins and FDA approved 3410 anti-viral drugs. On the other hand, a good number of authors independently proposed several sets of host genomic biomarkers (target proteins) associated with SARS-CoV-2 infections [26–46]. Some of the articles also suggested few candidate-drugs for the treatment against SARS-CoV-2 infections. But so far none of them compared their results either computationally or experimentally with each other. We reviewed their articles and found that there was not any set of drug targets/agents that are commonly proposed in all of articles. This type of dissimilarities may be due to the computational and/or environmental variations. Obviously, a question may be raised, how a particular vaccine or a drug can be effective commonly for all peoples around the world. Therefore, in this study, our main objectives are (1) computational identification of host genomic biomarkers (drug target proteins) for SARS-CoV-1 infections highlighting their functions, pathways, regulators and associated comorbidities; (2) identification of repurposable drugs for SARS-CoV-1 infections and (3) validation of the proposed candidate-drugs against different variants of SARS-CoV-2 infections. The working flowchart of this study is displayed in **Fig 1.**

## 2 Materials and methods

### 2.1 Data sources and descriptions

We used both original data and metadata associated with SARS-CoV infections to reach the goal of this study as described in subsections 2.1.1–2.1.2.

#### 2.1.1 Collection of host microarray gene-expression profiles to explore drug target proteins

We collected gene expression profiling of peripheral blood mononuclear cells (PBMC) with SARS-CoV-1 infection as original data to explore host genomic biomarkers (drug target proteins). The dataset was downloaded from the affymetrix human HG-Focus target array platform under the NCBI Gene Expression Omnibus (GEO) data repository (https://www.ncbi.nlm.nih.gov/geo/query/acc.cgi?acc=GSE1739) with the accession number GSE1739 [47]. It consisted of 14 samples, where the number of SARS cases was 10 and the number matched control sample was 4. This dataset was first analyzed by Reghunathan et al. [2005] to understand the host response to SARS-CoV-1 infection from gene expression level [48].

#### 2.1.2 Collection of already published drug agents and target proteins as metadata to explore commonly effective drugs

We collected SARS-CoV-2 3CL protease-guided top listed 90 drugs out of 3410 FDA approved anti-viral drugs published by Beck et al. [25] as the meta drug agents to explore few top ranked host transcriptome-guided drugs against SARS-CoV-2 infections by molecular docking with our proposed receptor proteins. The 3D structures of 90 FDA-approved drugs (**S1 Table**) were downloaded from PubChem database [49]. To evaluate the proposed drugs by cross-validation with the already available target proteins (published), we reviewed 22 different articles [26–46] associated with SARS-CoV-2 infections and collected 193 target proteins.

### 2.2 Integrated bioinformatics approaches

To explore genomic biomarkers highlighting their functions, pathways, regulatory factors and associated comorbidities, we utilized statistical r-package LIMMA, online databases STRING, GO, KEGG and DisGeNET, online tools NetworkAnalyst, Kaplan-Meier (KM) plotter and Cytoscape. To explore genomic biomarker-guided repurposable drugs, we performed molecular docking analysis by using offline tools (Discovery Studio Visualizer, USCF Chimera, PyMol and Autodock vina in PyRx) and online tool Protein-Ligand Interaction Profiler (PLIP) web service. The detail procedure is discussed below in the subsections 2.2.1–2.2.7.

#### 2.2.1 Identification of differential gene expressions (DEGs)

The linear models for microarray (LIMMA) approach [50] are widely used to identify differentially expressed genes (DEGs) between two conditions [20–24]. Therefore, we considered the LIMMA approach to identify DEGs between SARS-CoV and controls samples from microarray gene expression profiles. In LIMMA approach, the *p*-value is calculated by using the modified *t*-statistic to test the significance of differential gene expressions between two conditions. The *p*-values are then adjusted for multiple testing using the procedure of Benjamini and Hochberg. Then we considered the *g*th gene (*g* = 1,2,…, G) as a differentially expressed gene (DEG) between case and control groups if its adjusted *p*_*g*_*-*value<0.05 along with |log_2_(aFC_*g*_)|> 1 by controlling the false discovery rate (FDR) at 5%, otherwise, it was considered as equally expressed gene (EEG). The *g*th gene was considered as upregulated if the adjusted *p*_*g*_*-*value<0.05 along with log_2_(aFC_*g*_)> 1.5; otherwise, it is said to be downregulated. Here aFC is defined as ${aFC}_{g}={\bar{x}}_{g}/{\bar{y}}_{g}$ (the fold change of ${\bar{x}}_{g}$ with respect to ${\bar{y}}_{g}$), where ${\bar{x}}_{g}$ and ${\bar{y}}_{g}$ are the averages of expressions of case and control groups with respect to *g*th gene, respectively. For example, a change from ${\bar{y}}_{g}=3$ to ${\bar{x}}_{g}=9$ produces aFA_g_ = 3 which is referred to as a "3-fold upregulated in average". Similarly, a change from ${\bar{y}}_{g}=9$ to ${\bar{x}}_{g}=3$ produces aFA_g_ = 1/3 which is referred to as a "3-fold downregulated in average". The volcano plot and hierarchical clustering were used to display the significant down-regulated and up-regulated genes and this plot was implemented on the basis of the Designer canvas package [51].

#### 2.2.2 Protein-protein interactions (PPI) network analysis of DEGs

Protein–protein interactions (PPIs) are the physical magnetism of two or more protein molecules that occur due to biochemical reactions steered by hydrogen bonding, electrostatic forces and the hydrophobic effect. Generally a protein cannot work without interaction with one or more other proteins. The PPIs contribute to the formation of larger protein complexes for performing a specific task [52]. It carries out many molecular functions and biological processes including protein function, cell-to-cell interactions, metabolic and developmental control, disease incidence, and therapy design. A PPI network is represented as an undirected graph, where nodes and edges indicate proteins and their interactions, respectively. A node having the largest number of significant interactions/connections/edges with other nodes is considered as the top ranked hub-protein. Therefore, the PPI network analyses of DEGs are now widely using to explore HubGs/proteins. In this study, the PPI network of DEGs was constructed through the STRING database [53] to detect HubGs. The NetworkAnalyst [54] and Cytoscape 3.8.0 were used to visualize and perform topological analyses of PPI network. As a cutoff value in PPI, medium confidence score 900 was used. Topological degree of measurement (> 25) is used to identify the HubGs within the PPI network.

#### 2.2.3 Functional and pathway enrichment analysi*s* of DEGs

Gene ontology (GO) functional and Kyoto encyclopedia of genes and genomes (KEGG) pathway enrichment/annotation/over-representation analysis is a widely used approach to determine the significantly annotated/enriched/over-represented functions/classes/terms (biological processes (BP), molecular functions (MF) and cellular components (CC)) and pathways by the identified DEGs. The BP is a change or complex of changes during the granularity period of the cell that is mediated by one or more gene products for different biological objectives. The MFs are the biochemical activities of gene products. The CC is a place in a cell in which a gene product is active. KEGG pathway is a collection of experimentally validated pathway maps representing our knowledge of the molecular interaction, reaction and relation networks for metabolism, cellular processes, genetic information processing, organismal systems, environmental information processing, human diseases and drug development. We performed HubGs functional and pathway enrichment analysis using the NetworkAnalyst tool with GO and KEGG databases [54, 55]. A Fisher exact test with the cut-off adjusted *p*-value <0.05 was applied to determine the statistical significance of the functional enrichment analysis. Again we performed DEGs for the same analysis (functional and pathway enrichment) using more three popular tool DAVID [56], EnrichR [57] and Metascape [58] with GO and KEGG databases. And finally we suggested common significant enriched term (i.e. which terms are statistically significant and enriched in the every tools) for the reliability of the results.

#### 2.2.4 Regulatory network analysis of HubGs

Transcriptional and posttranscriptional regulations of genes play the vital roles in a lot of cellular and molecular functions and biological processes. A gene regulatory network (GRN) shows molecular regulators that interact with each other to control the gene expression levels of mRNA and proteins. Transcription factors (TFs) and microRNAs (miRNAs) are considered as the most important molecular regulators of genes. A transcription factor (TF) is a protein that binds to a specific DNA region (promoter/enhancer) and regulates gene expression by promoting or suppressing transcription. TFs are considered as the main players in GRN. A miRNA is a small single-stranded non-coding RNA molecule (containing about 22 nucleotides) that works in RNA silencing and post-transcriptional regulation of gene expression. There are up to 1600 TFs and 1900 miRNAs in the human genome. The TFs and HubGs/proteins interaction network is considered as an undirected graph, where nodes indicate TFs or HubGs and edges represents interactions between TFs and HubGs, respectively. A TF-node having the largest number of interactions/connections/edges with HubGs is considered as the top ranked hub-TF regulator of HubGs. We performed the TFs-HubGs interactions network analysis through JASPAR database [59] to determine the key TFs associated with HubGs. The key miRNAs which regulate the HubGs in the post-transcriptional level were identified by the analysis of miRNAs-HubGs interactions with the TarBase [60] and miRTarBase [61] databases and the interactions were regenerate via NetworkAnalyst [54]. As the regulator of the identified HubGs, the top miRNAs were selected according to highest topological degree. Again we performed the same analysis (TFs and miRNAs) using another popular tool EnrichR [57] with JASPAR and miRTarBase databases. And finally we proposed common enriched TFs and miRNAs (i.e. which terms are enriched the both tools) for the reliability of the results. Chemical-HubGs interactions were analyzed from the comparative toxicogenomic database (CTD) to deal with the growing demand in toxicogenomics [62].

#### 2.2.5 Association of HubGs with comorbidities

To investigate the association of HubGs with other diseases, we performed diseases versus HubGs interaction network analysis by using the NetworkAnalyst tool [54] based on DisGeNET [63] database. We also performed survival analysis based on the expression of HubGs with lung cancer patients by using SurvExpress [64] to investigate the association of hubGs with lung cancer, since SARS-CoV-2 samples were collected from lung tissue. The SurvExpress utilizes the log rank statistic to test the significance of association.

#### 2.2.6 Drug repurposing by molecular docking simulation

To propose *in-silico* validated efficient FDA approved repurposed drugs for the treatment against SARS-CoV infections, we employed molecular docking simulation between the drug target receptor proteins and drug agents. We considered our proposed hub-proteins (genomic biomarkers) and associated TFs proteins as the drug target receptor proteins and SARS-CoV-2-Spike proteome-guided top ranked 90 drugs out of FDA approved 3410 anti-viral drugs suggested by Beck et al. [25] as meta drug agents or ligands for docking analysis. The molecular docking simulation requires 3-Dimensional (3D) structures of both receptor proteins and candidate-drugs. The 3D structures of FDA approved 90 drugs were downloaded from PubChem database [49] seen in the **S1 Table**. Interactions between drugs and proteins were calculated based on their binding affinities (kcal/mol). AutoDock Vina was used for molecular docking and virtual screening of drug agents by computing the binding affinities (kcal/mol) [65, 66] between hub proteins and 100 FDA-approved drugs. The exhaustiveness parameter was set to 8. The USCF Chimera and Discovery Studio Visualizer 2019 were used to generate the structure and to visualize the 2D protein- ligand interactions [67, 68]. Finally, we cross validated our docking results through PatchDock webser docking tool [69]. Drug Bank is a specialized database for drug molecular information, mechanisms of action and drug-target information for >10 000 drugs. Drug Bank [70] and NCBI-PubChem database [49] was used to collect the information of drugs such as the mechanism of action and therapeutic indications. Let *A*_*ij*_ denotes the binding affinity between *i*th target protein (*i* = 1, 2, ….., *m*) and *j*th drug agent (*j* = 1, 2, ….., *n*). Then target proteins are ordered according to the descending order of row sums $\sum_{j=1}^{n}A_{ij}$, *j* = 1,2,…, *m*, and drug agents are ordered according to the descending order of column sums $\sum_{i=1}^{m}A_{ij}$, *j* = 1,2,…, *n*, to select the top ranking few drug agents as the candidate-drugs. Then we validated the proposed repurposed drugs by molecular docking simulation with the top ranked receptor proteins associated with SARS-CoV-2 infections that were obtained by the literature review. To select the top listed receptor proteins associated with SARS-CoV-2 infections, we reviewed 22 recently published articles [26–46] and selected the top listed 11 receptor proteins.

#### 2.2.7 Phylogenetic analysis of different variants of coronavirus sequences

To investigate the genetic evolutionary relationship among different variants of coronaviruses by phylogenetic tree analysis, we randomly selected SARS-COV, MERS-CoV and SARS-CoV-2 genome sequences available in GISAID (https://www.gisaid.org/) and the National Center for Biotechnology Information GenBank (https://www.ncbi.nlm.nih.gov/) platforms. All the sequences were aligned by the Neighbor-Joining method through the MEGA 11.0 multiple sequence alignment software [71]. The pairwise alignment parameters were considered as gap opening penalty 16, extension penalty 6.67, delay cutoff 35%, and transition weight of DNA 35%; weight matrix IUB with 1000 bootstrap replicates [72]. Nucleotide missing or fragmented data for every gene were erased. The breakpoints were detected using the phylogenetic incongruence among segments in sequence alignments using GARD.

## 3 Results

### 3.1 Identification of differential expression of genes (DEGs)

**Fig 2:**
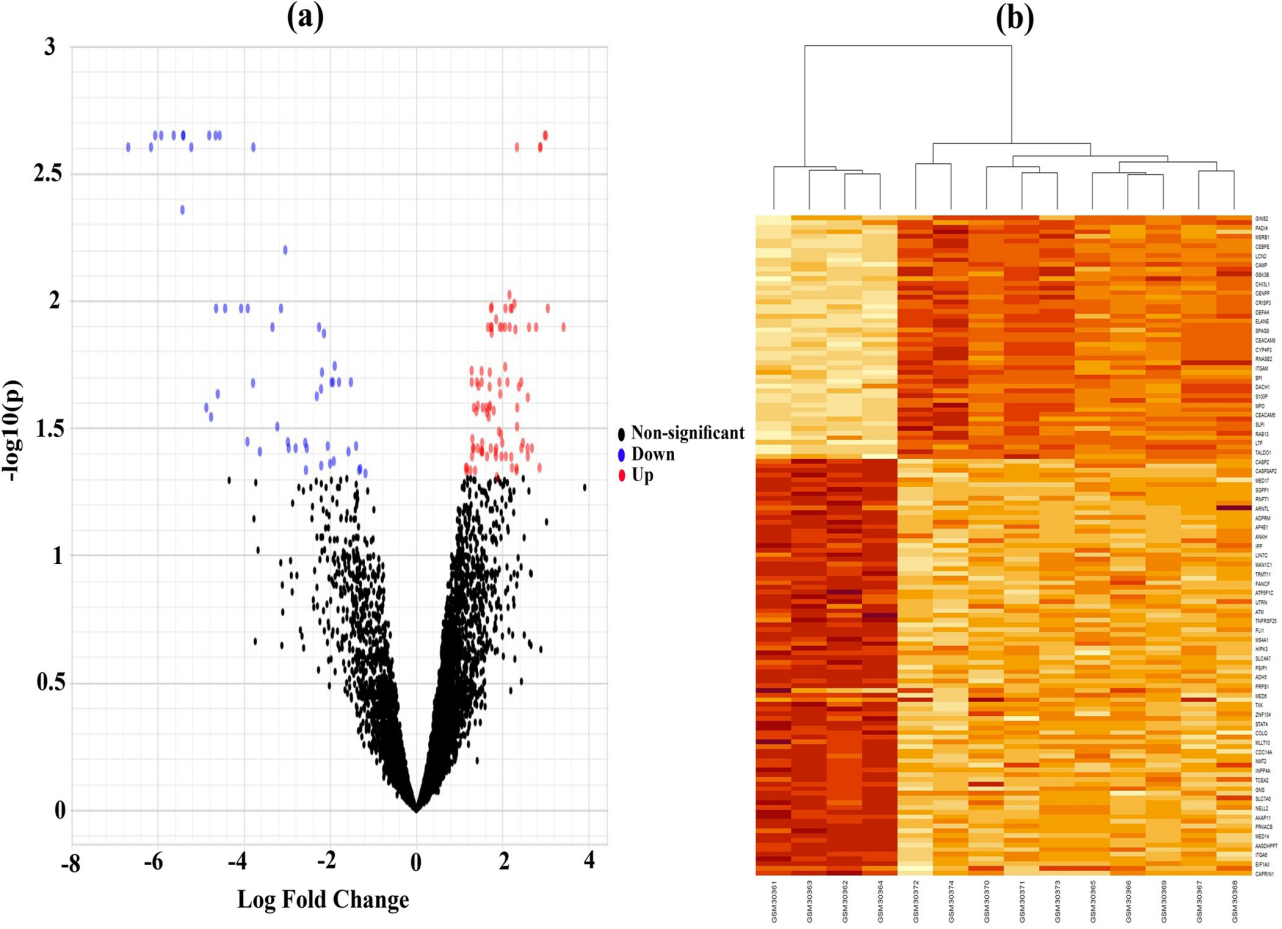
(a) Volcano plot of microarray data highlighting DEGs (blue: down regulated; red: up regulated; and black: insignificant), (b) Hierarchical clustering of DEGs to visualize up-regulated and down-regulated genes.

To identify DEGs between SARS-CoV-1 infected and control samples, we analyzed a publicly available gene expression dataset (GSE-1739) by using statistical LIMMA approach and identified 141 DEGs, where 87 DEGs were up-regulated and 51 DEGs were down-regulated. A volcano plot was constructed to display the status of all genes simultaneously, where red color indicate significant up-regulated, blue color are significant down-regulated genes and black color indicate insignificant genes. The DEGs were selected with the threshold of adjusted *p*-values <0.05 and the absolute of fold change values >1.0 **(see Fig 2A)**. We constructed Heatmap to observe the performance of DEGs on clustering/classification of samples into case and control groups through the hierarchical clustering approach (see **Fig 2C**). We observed that DEGs were separated into up-regulated and down-regulated groups, and samples were separated into case and control groups properly **(see Fig 2B)**. We provided the up-regulated and down-regulated DEGs in **S2 Table** for further investigation by the other researchers.

### 3.2 Protein-protein interactions (PPI) network analysis of DEGs

**Fig 3:**
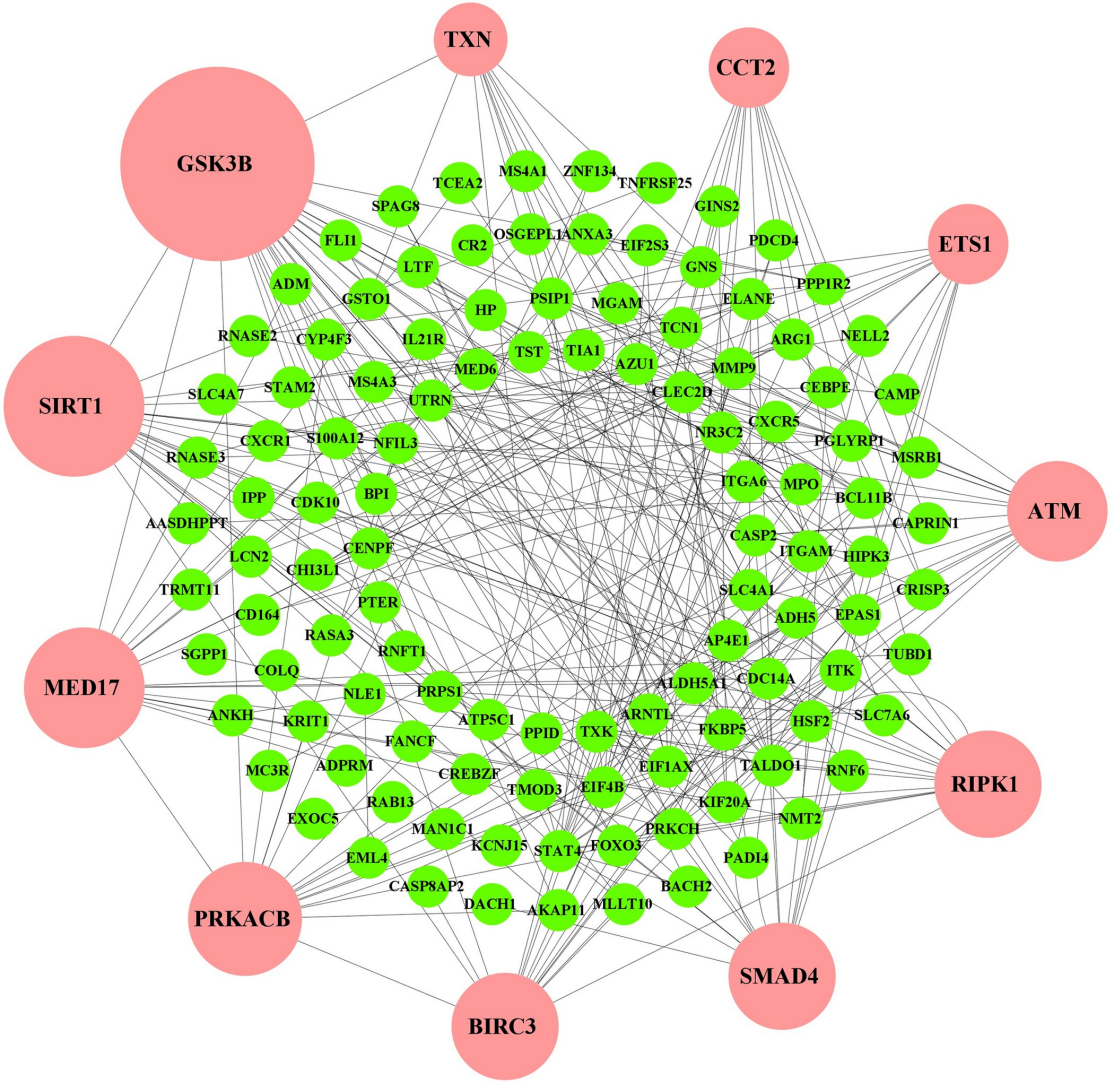
PPI network of DEGs. The larger nodes highlighted with pink color indicate the HubGs.

The PPI network of DEGs was reconstructed through the STRING database using the NetworkAnalyst tool in Cytoscape software platform. A topological exploration based on dual-metric measurements degree (>10) and betweenness were utilized to determine the highly representative DEGs/proteins those are also known as hub-DEGs/proteins. The top 11 HubGs (SMAD4, GSK3B, SIRT1, ATM, RIPK1, PRKACB, MED17, CCT2, BIRC3, ETS1 and TXN) were highlighted using the larger nodes with pink color **(Fig 3 and S3 Table)**. Among the top 11 HubGs, GSK3B and TXN are down regulated and the rest 9 HubGs are upregulated. The selected 11 HubGs were considered as the key genomic biomarkers (drug target proteins).

### 3.3 Functional and pathway enrichment analysis of HubGs

The GO functional and KEGG pathway enrichment analyses of DEGs reflected the biological processes (BPs), molecular functions (MFs), cellular components (CCs) and pathways that are highly linked with the COVID-19 infection (**Table 1**). Among the enriched Go terms (BPs, MFs and CCs) with DEGs in 4 different databases (NetworkAnalyst, DAVID, EnrichR and Metascape), 10 BPs (apoptotic signaling pathway, immune response, interleukin-8 production, leukocyte differentiation, regulation of angiogenesis, regulation of apoptotic process, regulation of cytokine production, regulation of programmed cell death, response to hypoxia and response to oxidative stress), 2 MFs (transcription factor binding, oxidoreductase activity) and a CC term (secretory granule) were enriched by involving HubGs. Among the enriched KEGG pathways, 3 pathways (transcriptional mis-regulation in cancer, pathways in cancer, chemokine signaling pathway) were also enriched by involving HubGs.

**Table 1:** Significantly enriched GO terms and KEGG pathways with DEGs by involving HubGs in four different databases that are involved in the pathogenetic processes of SARS-CoV infections (p-value <0.05).

	Involved HubGs by NetworkAnalyst	Involved HubGs by David	Involved HubGs by EnrichR	Involved HubGs by Metascape
GO: BP				
Apoptotic signaling pathway	RIPK1, SIRT1, ATM	GSK3B, SIRT1, RIPK1, ATM	GSK3B, RIPK1	ATM, GSK3B, RIPK1, SIRT1
Immune response	BIRC3, ETS1, RIPK1, SIRT1, TXN	ETS1, RIPK1, PGLYRP1, PRKACB, SIRT1, BIRC3		
Interleukin-8 production	RIPK1	RIPK1	RIPK1	RIPK1
Leukocyte differentiation	ATM	RIPK1, ATM, SIRT1	SIRT1	ATM, SIRT1
Regulation of angiogenesis	ETS1	ETS1, SIRT1	ETS1, SIRT1	ETS1, SIRT1
Regulation of apoptotic process	BIRC3, GSK3B, ETS1, RIPK1, SIRT1, ATM	GSK3B, ETS1, SIRT1, RIPK1, BIRC3	GSK3B, SIRT1, RIPK1, ATM, BIRC3	ATM, SMAD4, RIPK1, SIRT1
Regulation of cytokine production	BIRC3, SMAD4, RIPK1	SMAD4, RIPK1, BIRC3		RIPK1, SIRT1
Regulation of programmed cell death	BIRC3, GSK3B, ETS1, RIPK1, SIRT1, ATM	GSK3B, ETS1, SIRT1, RIPK1, ATM, BIRC3	RIPK1, ATM, SIRT1, BIRC3	ATM, SMAD4, RIPK1, SIRT1
Response to hypoxia	ETS1, SMAD4, SIRT1, ATM	SMAD4, ATM, ETS1, SIRT1	SIRT1	ATM, SMAD4, SIRT1
Response to oxidative stress	ETS1, SIRT1	TXN, ETS1, SIRT1, RIPK1		TXN, RIPK1, SIRT1
**GO: MF**				
Transcription factor binding	ETS1, GSK3B, MED17, SIRT1	GSK3B, SMAD4, ETS1, SIRT1, MED17	SIRT1	ETS1, GSK3B, SMAD4, MED17, SIRT1
Oxidoreductase activity, acting on a sulfur group of donors	TXN	TXN	TXN	
**GO: CC**				
Secretory granule	CCT2		CCT2	CCT2
**KEGG pathway**				
Transcriptional misregulation in cancer	BIRC3, ATM	ATM	ATM, BIRC3	BIRC3, ATM
Pathways in cancer	BIRC3, ETS1, GSK3B, PRKACB, SMAD4	GSK3B, SMAD4, ETS1, PRKACB, BIRC3	GSK3B, SMAD4, ETS1, PRKACB, BIRC3	BIRC3, ETS1, GSK3B, SMAD4, PRKACB
Chemokine signaling pathway	GSK3B, PRKACB	GSK3B, PRKACB	GSK3B, PRKACB	GSK3B, PRKACB

### 3.4 Regulatory network analysis of HubGs

**Fig 4:**
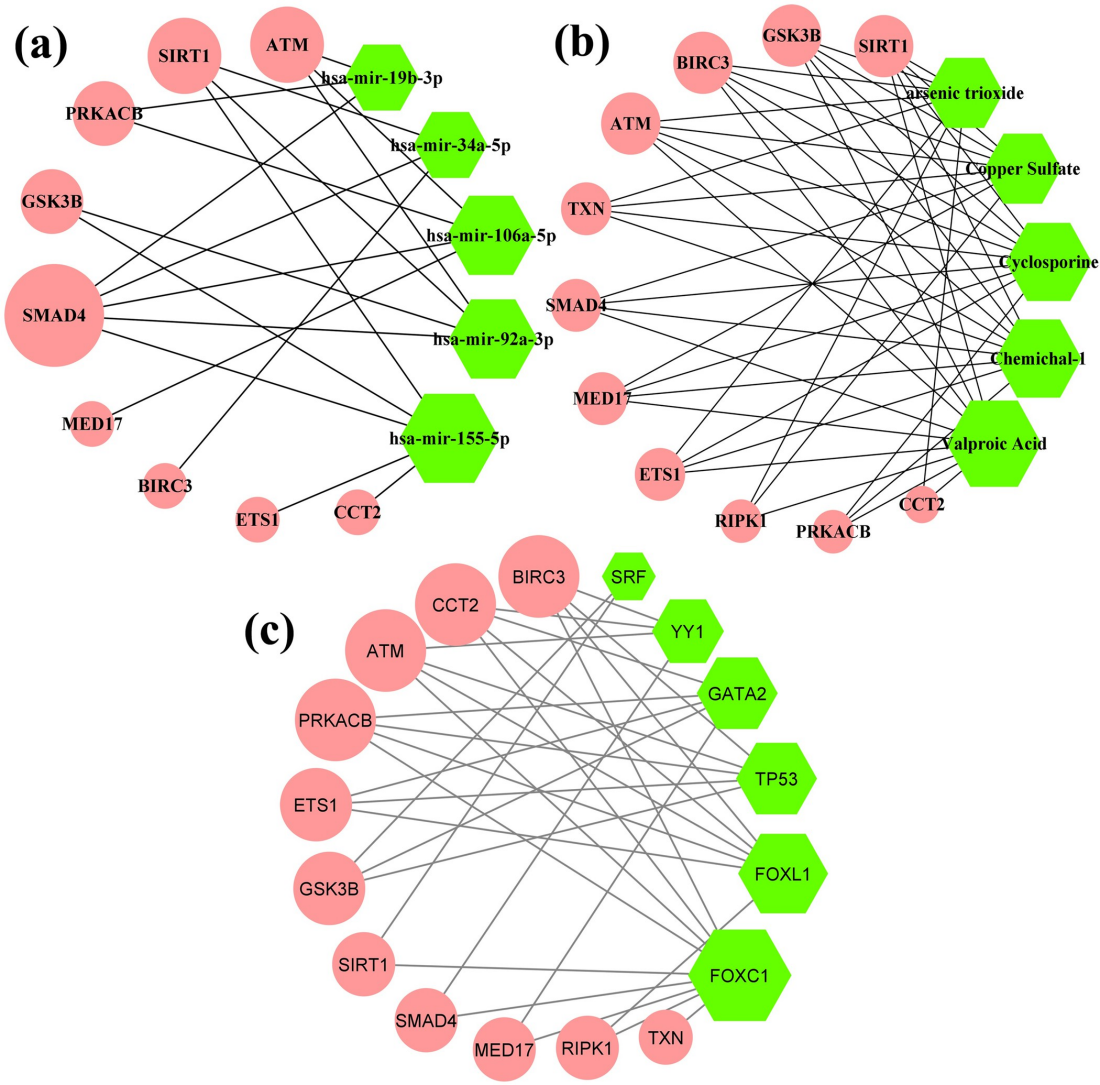
(a) miRNAs-HubGs interaction network based on TarBase and miRTarBase databases (b) Chemicals-HubGs interaction network based on comparative toxicogenomics database; where, Chemichal-1: (6-(4-(2-piperidin-1-ylethoxy)phenyl))-3-pyridin-4-ylpyrazolo(1,5-a)pyrimidine. (c) TFs-HubGs interaction network based on JASPAR database.

We performed miRNAs-HubGs, Chemicals-HubGs and TFs-HubGs interaction networks and enrichment analyses to identify the key regulators of HubGs at transcriptional and posttranscriptional levels by using two popular tools NetworkAnalyst and EnrichR. We constructed undirected interaction networks between regulatory factors and HubGs as shown in **Fig 4(A)–4(C)**. In these networks, HubGs were represented by round nodes with pink color and key regulatory factors were represented by rounds nodes with green color, where larger number of connectivity’s were represented by the larger node sizes as before. Both the NetworkAnalyst and EnrichR (**Fig 4** and **S4 Table)** showed 5 miRNAs (hsa-mir-92a-3p, hsa-mir-155-5p, hsa-mir-106b-5p, hsa-mir-34a-5p and hsa-mir-19b-3p) as the key regulators of HubGs at post-transcriptional levels, 6 chemicals (Valproic Acid, Cyclosporine, (6-(4-(2-piperidin-1-ylethoxy)phenyl))-3-pyridin-4-ylpyrazolo(1,5-a)pyrimidine, Copper Sulfate and arsenic trioxide as the key regulators of HubGs at transcriptional and posttranscriptional levels, and 6 TFs (FOXC1, GATA2, YY1, FOXL1, TP53 and SRF) as the key regulators of HubGs at transcriptional levels.

### 3.5 Association of HubGs with comorbidities

**Fig 5:**
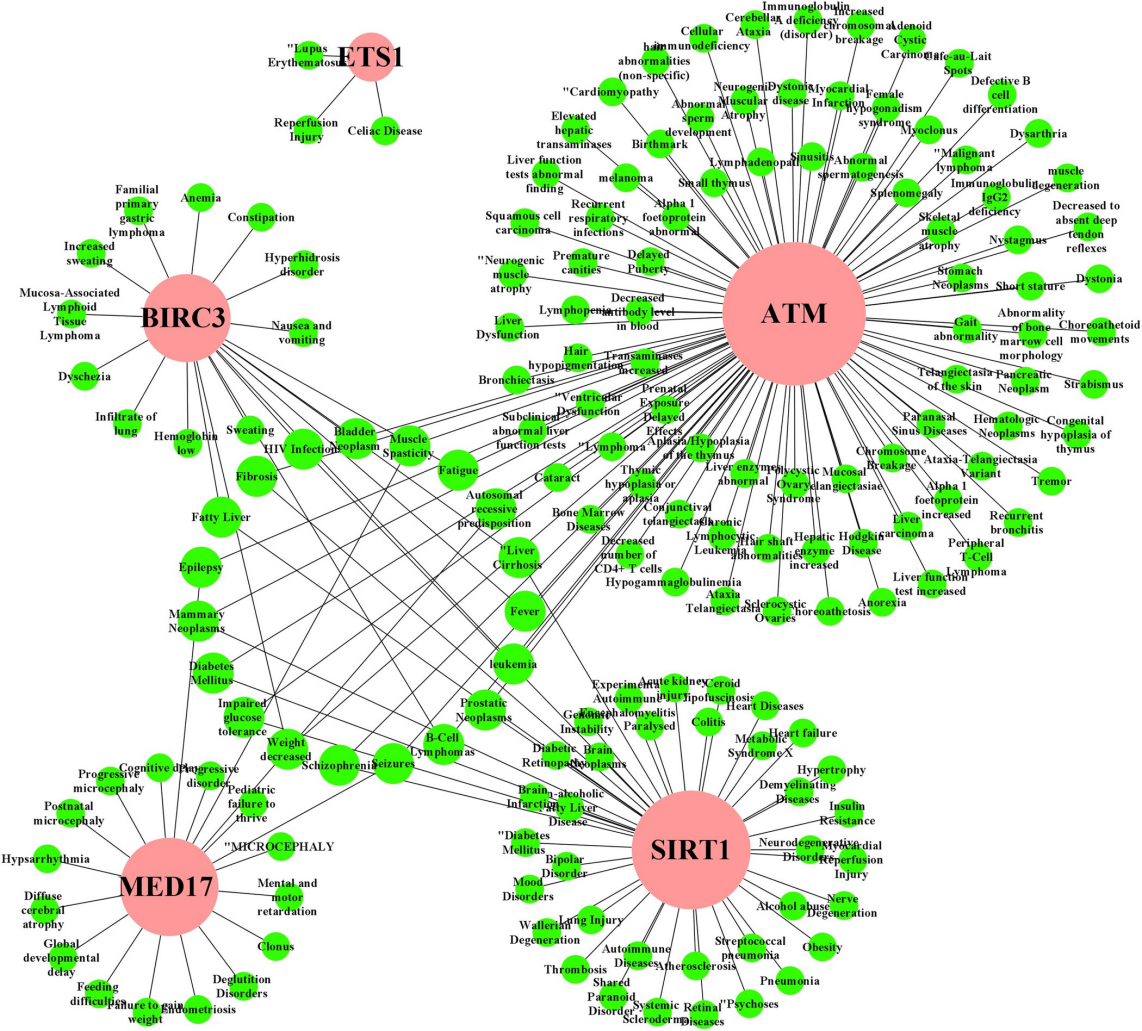
Identification of comorbidities that may be influenced genetically by the SARS-CoV-1 infections. The HubGs versus disease interaction network, where circle-shape with pink color indicates HubGs and circle-shape with other color indicates different diseases/comorbidities.

To assess the link of our predicted key genomic biomarkers with other diseases, we performed their interaction network analysis. **Fig 5** shows the disease versus HubGs interaction network analysis results. We observed that ATM gene is associated with 89 diseases including respiratory infections, liver carcinoma, bronchiectasis, diabetes, cellular immunodeficiency, fever, leukemia, fibrosis; the SIRT1 was associated with 41 diseases including lung injury, autoimmune diseases, liver cirrhosis, HIV infections, diabetes, heart diseases; the HubGs MED17, BIRC3 and ETS1 were linked with 20, 19 and 3 diseases, respectively including infiltrate of lung, anemia, liver cirrhosis, fever, reperfusion injury, hypsarrhythmia; all these are also considered as the severe comorbidities for SARS-COV-2 infections (see **S5 Table** for more details).

**Fig 6:**
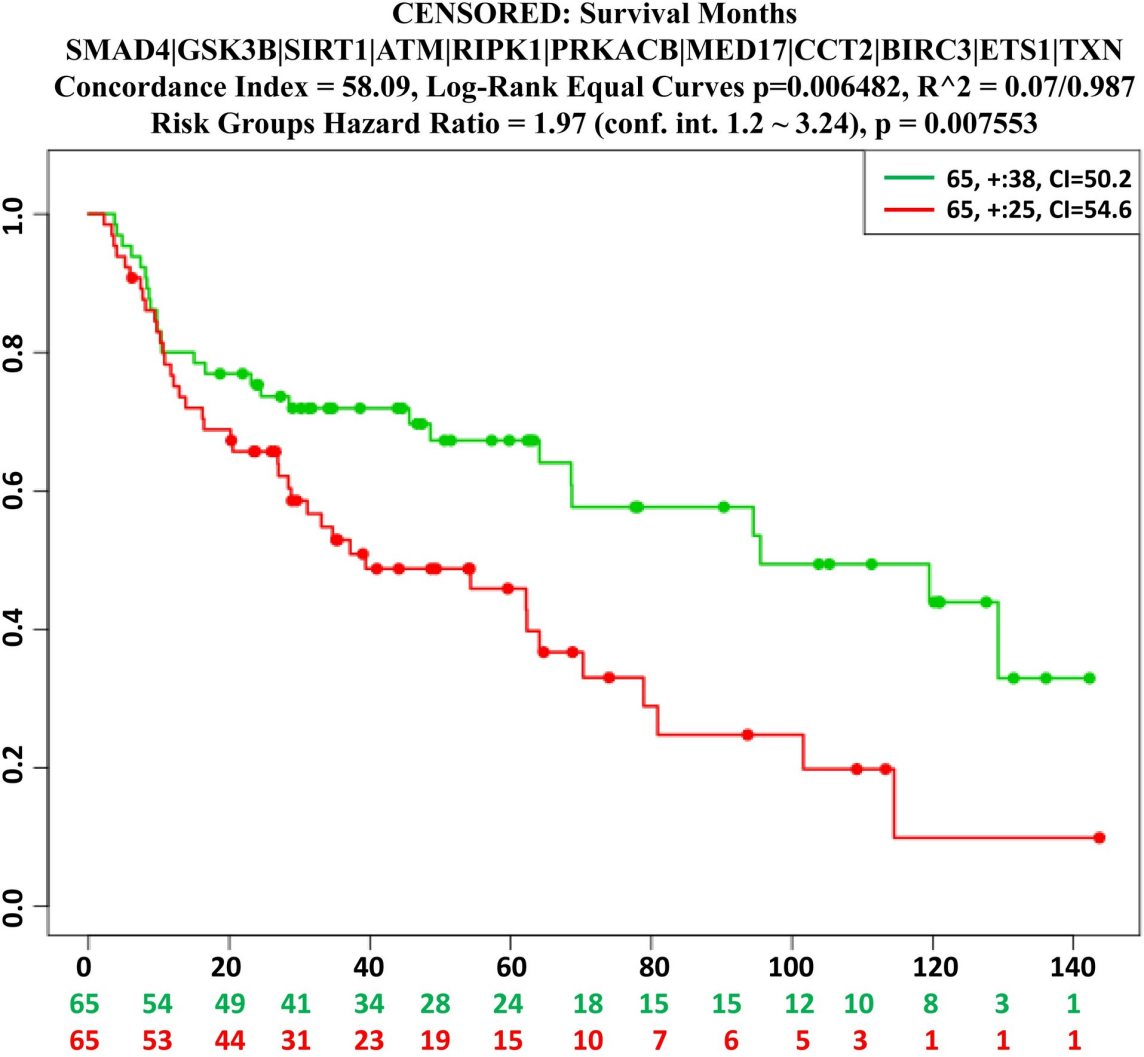
The multivariate survival curves of lung cancer patients based on hub-DEGs.

We also performed multivariate survival analysis based on lung cancer data with HubGs to assess their connection, since lung diseases are considered as the major risk factors of coronavirus infections. We observed the significant difference between the low and high risk group in survival probability (see **Fig 6)**, which indicates that HubGs are significantly associated with lung cancer.

### 3.6 Genetic relationship among SARS-CoV-1, MERS-CoV and SARS-CoV-2

**Fig 7:**
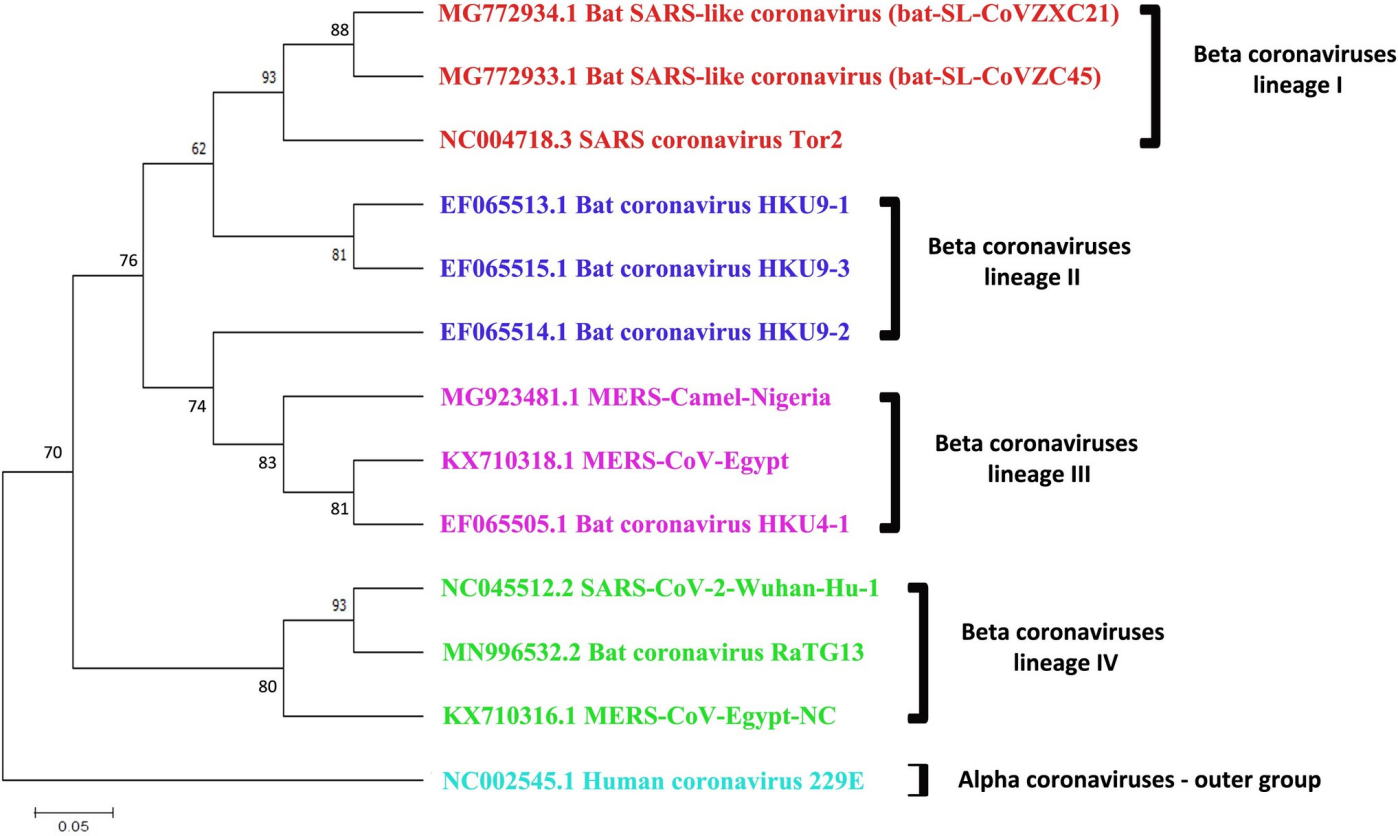
Phylogenetic analysis of SARS-CoV, MERS-CoV and SARS-CoV-2 (COVID-19) based on Neighbor-Joining method. SARS-CoV and MERS-CoV highly pathogenic beta coronaviruses along with SARS-like bat coronaviruses closely linked to SARS-CoV-2. Number at nodes indicates support for bootstrap (100 replicates), and the bar of scale indicates the average number of substitutions per location. The alpha coronavirus HCoV-229E sequence were considered as the out-group.

According to some recent studies, the genomic structures of SARS-CoV-2 are nearly identical to those of SARS-CoV-1 [8–11, 13–15]. To test this similarity, we built a phylogenetic tree using randomly chosen beta SARS-CoV, MERS-CoV, and SARS-CoV-2 genome sequences (**Fig 7**). The tree was divided into four groups (Cluster-I, Cluster -II, Cluster -III, and Cluster -IV). A whole-genome comparison of SARS-CoV-2 with other coronaviruses revealed 81%, 88%, and 83% similarity to bat-coronaviruses HKU9-1 to 3, bat-SL-CoVZXC21, and MERS-CoV, respectively. Interestingly, a notable 93% sequence similarity was discovered between the bat CoV RaTG13 (Rhinolophus affinis) and SARS-CoV-2 (Lineage IV) [73]. Further finding into host selection demonstrated that the bat CoV RaTG13 is an inner joint neighbor, with MERS-CoV-Egypt being the closest relatives. The lineage II, on the other hand, revealed a long branch between SARS-CoV-2 and the close relatives bat HKU9-1, HKU9-2, and HKU9-3 with sequence similarity less than 90%, implying that these isolates may not be the direct ancestors of SARS-CoV-2. This suggests that another mammal may have delivered as an intermediate host from which the insertion was obtained. A related phenomenon was also observed in the cases of related MERS-CoV and SARS-CoV (from lineage III). Because most of SARS-outer CoV-2’s and inner joint neighbors have bats as their natural host, these findings suggest that bats would be the most suitable native hosts of SARS-CoV-2.

### 3.7 Drug repurposing by molecular docking

**Fig 8:**
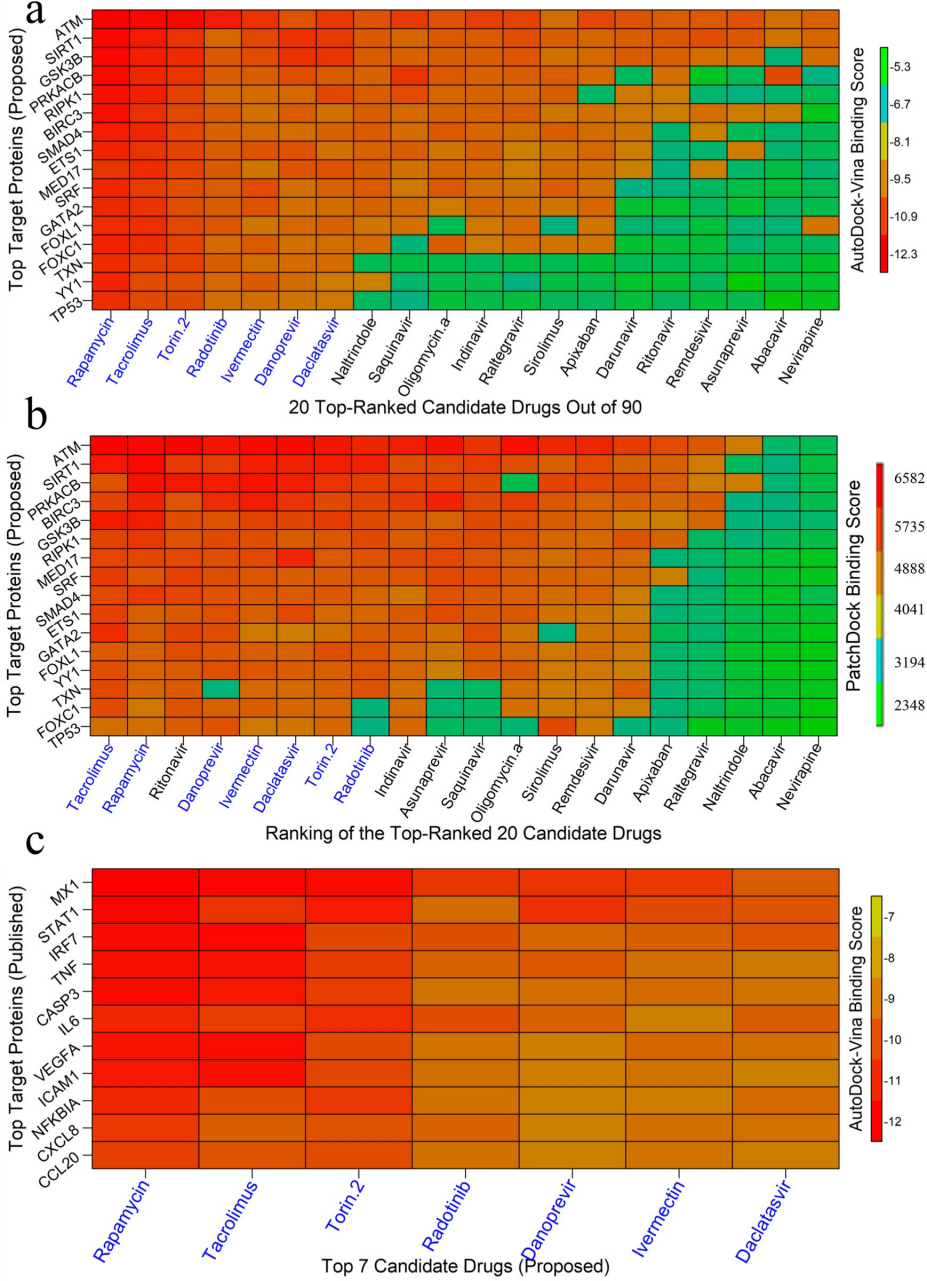
Molecular docking simulation results by AutoDock-Vina and PatchDock. Red colors indicated the strong binding affinities between target proteins and drug agents, and green colors indicated their weak bindings. (a) Image of binding affinity scores (computed by AutoDock-Vina) based on the top listed ordered 90 anti-viral drug agents in X-axis and ordered 17 target proteins (proposed) corresponding to SARS-CoV-1 in Y-axis. (b) Image of binding scores (computed by PatchDock) based on the ordered top-ranked 20 anti-viral drug agents (from 7a) in X-axis and ordered 17 target proteins corresponding to SARS-CoV-1 (proposed) in Y-axis. (c) Image of binding affinity scores based on the ordered proposed 7 candidate-drugs in X-axis and ordered more common 11 existing target proteins (published) corresponding to SARS-CoV-2 in Y-axis.

We considered our proposed 11 hub-proteins (genomic biomarkers) and its regulatory 6 key TFs proteins that are associated with SARS-CoV-1 infections as *m* = 17 drug target receptors and the SARS-CoV-2 3CL protease-guided top listed *n* = 90 drugs out of 3410 as drug agents/ligands as mentioned previously in the materials and method section 2.1.2. The 3D structures of target proteins (SIRT1, GSK3B, BIRC3, SMAD4, ATM, ETS1, RIPK1, PRKACB, TXN, TP53, GATA2, SRF, and YY1) were retrieved from Protein Data Bank (PDB) [74] with the PBD codes 5btr, 1i09, 2uvl, 1dd1, 5np0, 2nny, 6nyh, 4wb8, 1aiu, 1a1u, 5o9b, 1hbx, and 1ubd respectively and the rest MED17, FOXC1 and FOXL1 target proteins were downloaded from SWISS-MODEL [75] using UniProt ID of Q9NVC6, Q12948 and Q12952, respectively. The 3D structures of top ranked 90 FDA approved drug agents were downloaded from PubChem database [49] seen in the **S1 Table**. Molecular docking simulations were carried out between our proposed *m* = 17 targeted receptor proteins and *n* = 90 top listed drug agents to obtain binding affinities for each pair of target proteins and drug agents. Then we ordered the target proteins in descending order of row sums of the binding affinity matrix ***B*** = (*B*_ij_) and drug agents according to the column sums to select top ranked drug agents as the candidate-drugs. The **Fig 8A** displayed the image of binding affinity matrix $B^{*}=(B_{ij}^{*})$ corresponding to the ordered target proteins in Y-axis and ordered drug agents in X-axis. We observed that only top ranked 7 lead compounds (Rapamycin, Tacrolimus, Torin-2, Redotinib, Ivermectin, Danoprevir, Daclatasvir) produce significant binding affinities (≤ -7.0 kcal/mol) with all of our proposed 17 receptor proteins. To validate the AutoDock-Vina results by an another docking tool, we cross validated top-ranked 20 candidate-drugs (detected by AutoDock-Vina) by PatchDock web-server docking tool (Schneidman-Duhovny et al., 2005). The **Fig 8B** displayed the image of binding score matrix B^* = (B_ij^*) based on the ordered top-ranked 20 anti-viral drug agents (from **Fig 8A**) in X-axis and ordered 17 target proteins corresponding to SARS-CoV-1 (proposed) in Y-axis. From **Fig 8A and 8B**, we observed that 7 lead compounds Rapamycin, Tacrolimus, Torin-2, Redotinib, Ivermectin, Danoprevir and Daclatasvir are common within the top-ranked 8 compounds detected by AutoDock-Vina and PatchDock, respectively. Therefore, we considered these 7 lead compounds as the most probable candidate-drugs for SARS-CoV-1 infections.

To validate our proposed 7 candidate-drugs by molecular docking simulation with the already published target proteins associated with SARS-CoV-2 infections, we reviewed 22 articles on SARS-CoV-2 infections those provided HubGs (target proteins). We found total 193 HubGs that are published in those articles, but there was not any set of HubGs commonly published in those 22 articles **(see Table 2)**. We found only 11 HubGs (MX1, IRF7, NFKBIA, STAT1, IL6, TNF, CCL20, CXCL8, VEGFA, CASP3, ICAM1) in which each of them was common within 3–4 articles out of 22. It should be mentioned here that there was not any HubGs that was commonly published in more than 4 articles. We considered 11 target proteins corresponding to these 11 HubGs to validate our proposed drugs against SARS-CoV-2 infections by molecular docking simulation. The 3D structures of these 11 (MX1, IRF7, NFKBIA, STAT1, IL6, TNF, CCL20, CXCL8, VEGFA, CASP3, ICAM1) proteins were retrieved from Protein Data Bank (PDB) with codes 3szr, 2o61, 1nfi, 1bf5, 1il6, 1tnf, 2jyo, 1ikl, 1cz8, 1gfw and 5mza, respectively. Then we performed molecular docking simulations between our proposed 7 candidate and selected top ranked 11 target proteins (published) associated SARS-CoV-2 infections. Their binding affinity scores (kcal/mol) were visualized in **Fig 8C.** We observed that their binding affinity scores ranged between (-12.1 to -7) kcal/mol and average binding affinity scores were negatively larger than -9.5 kcal/mol which indicates the reasonably significant binding scores.

**Table 2:** Identification of top ranked target proteins associated with SARS-CoV-2 infections by literature review.

Articles	Hub-proteins
Wang et al. [26]	CXCL8, CXCL1, CXCL2, CCL20, CSF2
Gu et al. [27]	NFKBIA, C3, CCL20, BCL2A1, BID
Nan et al. [28]	ALB, CXCL8, FGF2, IL6, INS, MMP2, MMP9, PTGS2, STAT3, VEGFA
Gu et al. [29]	CDC20, NCBP1, POLR2D, DYNLL1, FBXW5, LRRC41, FBXO21, FBXW9, FBXO44, FBXO6
Sardar et al. [30]	HMOX1, DNMT1, PLAT, GDF1, ITGB1
Gu et al. [31]	FLOC, DYNLL1, FBXL3, FBXW11, FBXO27, FBXO44, FBXO32, FBXO31, FBXO9, CUL2
Xie et al. [32]	CXCL1, CXCL2, TNF, NFKBIA, CSF2, TNFAIP3, IL6, CXCL3, CCL20, ICAM1
Oh et al. [33]	GATA4, ID2, MAFA, NOX4, PTBP1, SMAD3, TUBB1, WWOX
Vastrad et al. [34]	TP53, HRAS, CTNNB1, FYN, ABL1, STAT3, STAT1, JAK2, C1QBP, XBP1, BST2, CD99, IFI35, MAPK11, RELA, LCK, KIT, EGR1, IL20, ILF3, CASP3, IL19, ATG7, GPI, S1PR1
Prasad et al. [35]	STAT1, IRF7, IFIH1, MX1, ISG15, IFIT3, OAS2, DDX58, IRF9, IFIT1, OAS1, OAS3, DDX60, OASL, IFIT2
Selvaraj et al. [36]	MYC, HDAC9, NCOA3, CEBPB, VEGFA, BCL3, SMAD3, SMURF1, KLHL12, CBL, ERBB4, CRKL
Satu et al. [37]	MARCO, VCAN, ACTB, LGALS1, HMOX1, TIMP1, OAS2, GAPDH, MSH3, FN1, NPC2, JUND, GPNMB, SYTL2, CASP1, S100A8, MYO10, IGFBP3, APCDD1, COL6A3, FABP5, PRDX3, CLEC1B, DDIT4, CXCL10, CXCL8
Taz et al. [38]	VEGFA, AKT1, MMP9, ICAM1, CD44
Moni et al. [39]	MX1, IRF7, BST2
Islam et al. [40]	BIRC3, ICAM1, IRAK2, MAP3K8, S100A8, SOCS3, STAT5A, TNF, TNFAIP3, TNIP1
Zhou et al. [41]	JUN, XPO1, NPM1, HNRNPA1
Ge et al. [42]	MMP13, NLRP3, GBP1, ADORA2A, PTAFR, TNF, MLNR, IL1B, NFKBIA, ADRB2, IL6
Aishwarya et al. [43]	IGF2, HINT1, MAPK10, SGCE, HDAC5, SGCA, SGCB, CFD, ITSN1, EHMT2, CLU, ISLR, PGM5, ANK2, HDAC9, SYT11, MDH1, CASP3, SCCPDH, SIRT6, DTNA, FN1, ARRB1, MAGED2, TEX264, VEGFC, HK2, TXNL4A, SLC16A3, NUDT21, TRA2B, HNRNPA1, CDC40, THOC1, PFKFB3
Saxena et al. [44]	STAP1, CASP5, FDCSP, CARD17, ST20, AKR1B10, CLC, KCNJ2-AS1, RNASE2, FLG
Tao et al. [45]	MAPK3, MAPK8, TP53, CASP3, IL6, TNF, MAPK1, CCL2, PTGS2
Zhang et al. [46]	CXCL10, ISG15, DDX58, MX2, OASL, STAT1, RSAD2, MX1, IRF7, OAS1
**List of hub-proteins in which each protein is common with at least 3 articles:** IL6, TNF, CCL20, CXCL8, VEGFA, ICAM1, IRF7, MX1, NFKBIA, STAT1, CASP3	
**List of hub-proteins in which each protein is common with at least 4 articles:** IL6, TNF	
**List of hub-proteins in which each protein is common with at least 5 articles:** N/A	

**Fig 9:**
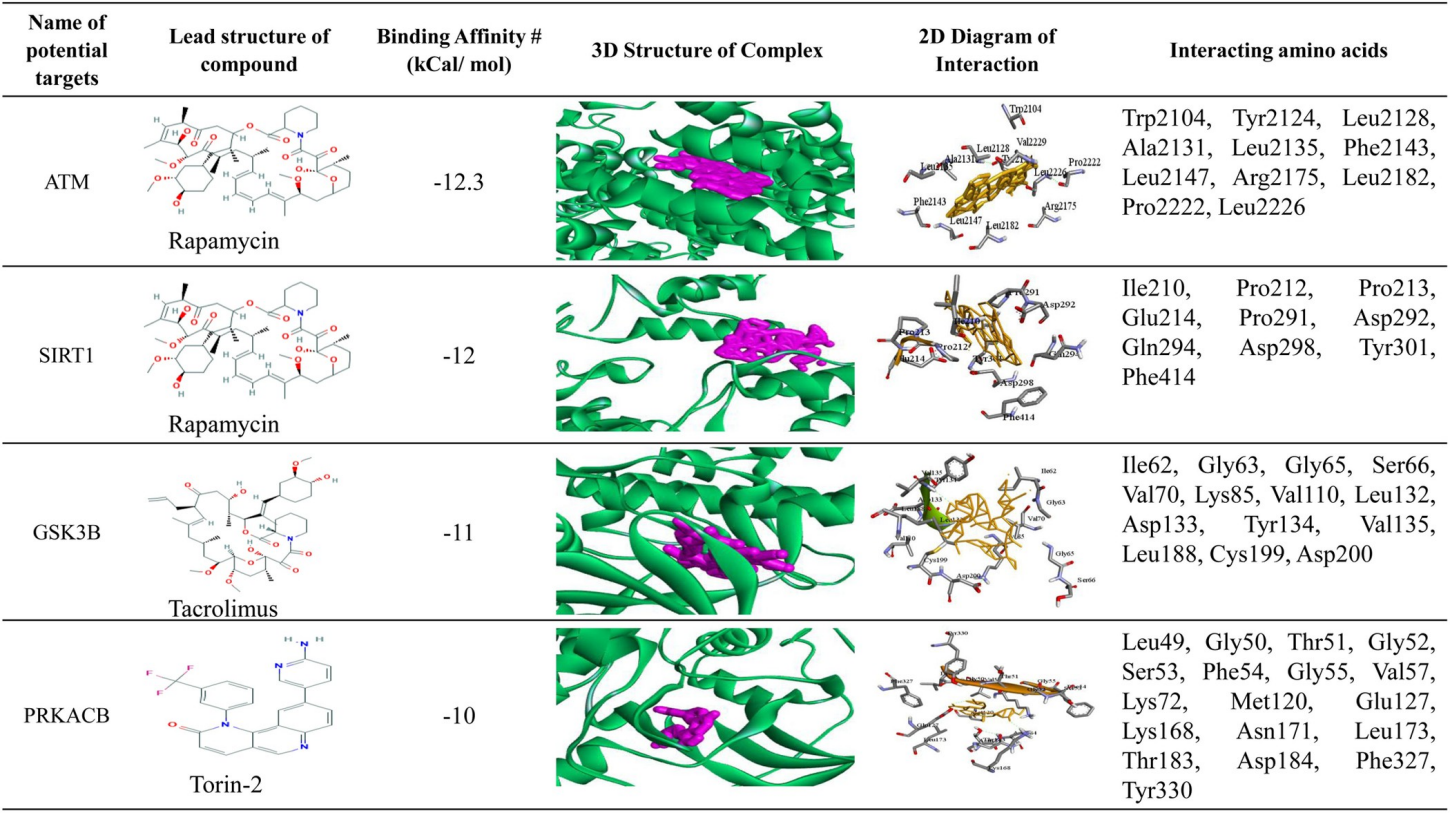
Top 4 potential targets and top 3 lead compounds (drugs) based on AutoDock-Vina docking results. Three candidate-drugs (Rapamycin, Tacrolimus, Torin-2) were selected based on their higher binding affinity scores. The 3D structure of hub protein with candidate-drugs is shown in the 4th column. The 2D Schematic diagram of hub protein with candidate-drugs interaction is given 5th column and neighbor residues (within 4 Å of the drug) are shown. Key interacting amino acids are shown in the last column.

**Fig 10:**
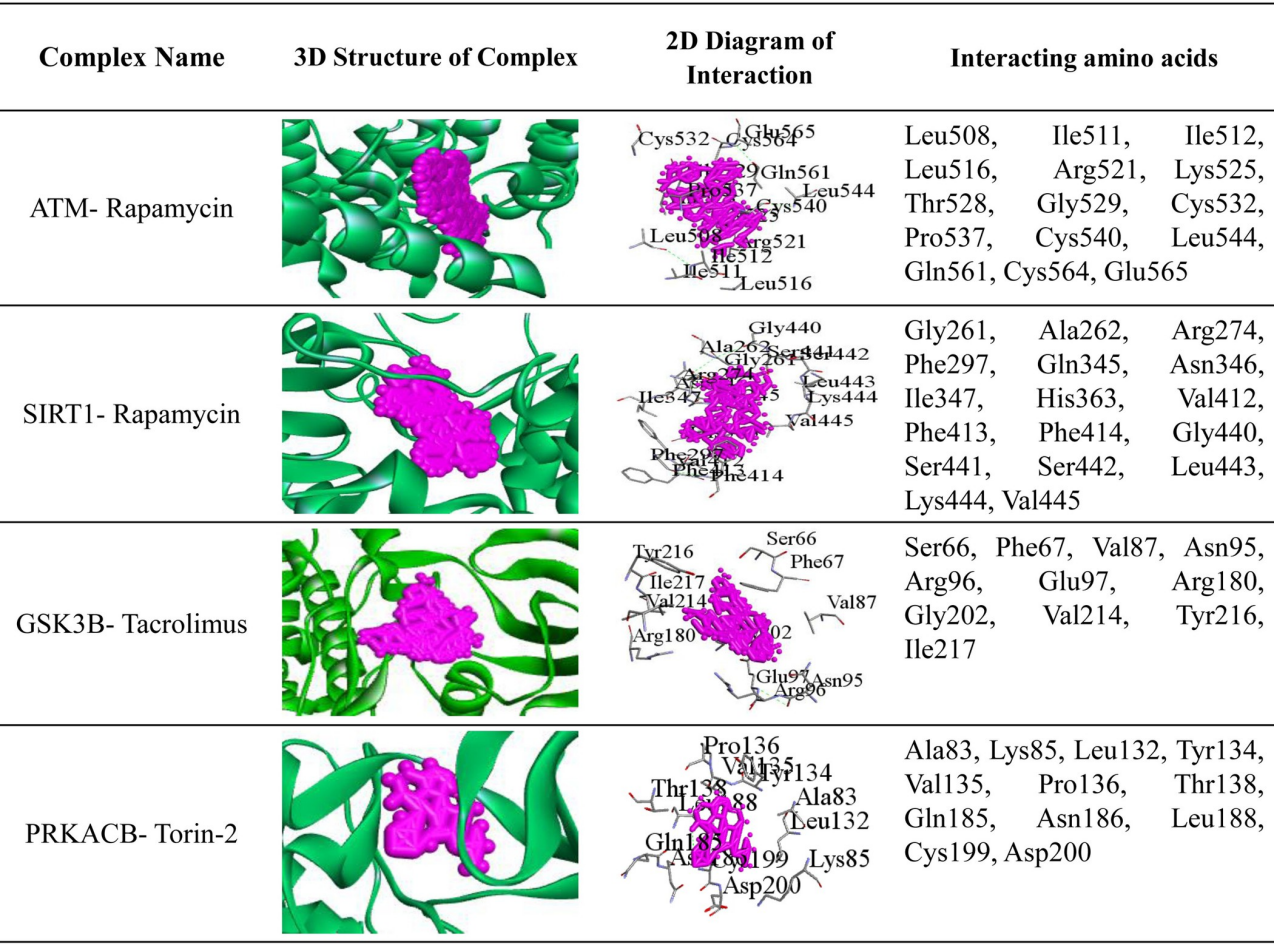
Top 4 potential targets and top 3 lead compounds (drugs) based on PatchDock docking results. The 3D structures of complexes are shown in 2nd column. The 2D Schematic diagrams of complexes are given in 3rd column and neighbor interacting residues (within 4 Å of the drug) are shown in the last column.

**Fig 9** shows binding pose of protein-ligand complex for top 4 potential target proteins and top 3 lead compounds (Rapamycin, Tacrolimus and Torin-2), where top 3 candidate-drugs (Rapamycin, Tacrolimus and Torin-2) were selected based on their higher binding affinity scores produced by AutoDock-Vina. The 3D structures of hub proteins with candidate-drugs are shown in the 4th column. The 2D Schematic diagram of hub protein with candidate-drugs interaction is given in the 5th column and neighbor residues (within 4 Å of the drug) are shown. Key interacting amino acids are shown in the last column. **Fig 10** and **Table 3** displayed the docking results corresponding to the most significant complexes between drugs and receptors produced by PatchDock. The **Table 4** shows the indications and mechanism of actions for the proposed repurposable drugs.

**Table 3:** PatchDock docking results corresponding to the most significant complexes between drugs and receptors.

Complex Name	Score^1^ (Geometric shape complementarity score)	Area^2^ (Approximate interface area of the complex)	ACE^3^ (Atomic contact energy)
ATM- Rapamycin	5880	734.00	-686.50
SIRT1- Rapamycin	4888	660.20	-393.97
GSK3B- Tacrolimus	4370	533.70	-272.37
PRKACB- Torin-2	4136	489.50	-160.09

^1^Geometric shape complementarity score.

^2^Approximate interface area of the complex.

^3^Atomic contact energy.

**Table 4:** Indications and mechanism of actions for the proposed repurposable drugs.

Drug bank or PubchemID	Proposed Drugs	Indication	Mechanism of action
DB00877	Rapamycin	To inhibit suppresses cytokine-driven T-cell proliferation, inhibiting the progression from the G1 to the S phase of the cell cycle. Activation of cytokines there by inhibiting cytokine production. It is bioactive only when bound to immunophilins. It is also a potent immunosuppressant.	Inhibitor
DB00864	Tacrolimus	Tacrolimus is an immunosuppressive drug whose main use is after organ transplant to reduce the activity of the patient’s immune system. It is also used in a topical preparation in the treatment of severe atopic dermatitis, severe refractory uveitis. It reduces peptidyl-prolyl isomerase activity by binding to the immunophilin FKBP-12 creating a new complex which inhibits calcineurin and T-lymphocyte signal transduction and IL-2 transcription.	Inhibitor
CID51358113	Torin 2	Torin 2 is an antiviral drug and DNA-damage response inhibitor as potent blocker of SARS-CoV-2 replication. Torin-2 also exhibits potent biochemical and cellular activity against PIKK family kinases including ATM, ATR and DNA-PK. Torin-2 also displayed marked anti-proliferative activity across a panel of cancer cell lines. Torin2 is used for treatment of cancer.	Inhibitor
DB11779	Danoprevir	Involvement in viral replication and suppressive effects on host response to viral infection, a promising new class of drugs for Novel Coronavirus Infectious Disease (COVID-19), Chronic Hepatitis C Virus (HCV) Infection	Inhibitor
DB09102	Daclatasvir	Treatment of chronic HCV (Chronic Hepatitis C Virus) genotype 1a/b or 3 infection	Inhibitor
DB00602	Ivermectin	For the treatment of intestinal strongyloidiasis, onchocerciasis and scabies.	Agonist
DB12323	Radotinib	Treatment of different types of cancer, most notably Philadelphia chromosome-positive (Ph+) chronic myeloid leukemia (CML) with resistance or intolerance of other Bcr-Abl tyrosine-kinase inhibitors.	Agonist

## 4 Discussions

In this study, we analyzed high throughput host gene-expression profiles for SARS-CoV-1 infections to identify key genomic biomarkers (drug target proteins) highlighting their functions, pathways, regulatory factors (TFs, miRNAs and chemicals), associated comorbidities and repurposable drugs for SARS-CoV-1 infections by using the integrated bioinformatics approaches. At first we identified 138 DEGs from host gene-expression profiles. Then we detected 11 HubGs as genomic biomarkers (SMAD4, GSK3B, SIRT1, ATM, RIPK1, PRKACB, MED17, CCT2, BIRC3, ETS1 and TXN) by the PPI network analysis of 138 DEGs (**Fig 3, S3 Table).** These 11 genomic biomarkers were treated as the key players for signal transduction during disease development. In particular, the key gene GSK3B may lead to the viral replication, initiation of oxidative stress, and inflammation during SARS-COV-2 infection [76, 77]. Recent studies also demonstrate that GSK3B is a putative therapeutic target to combat the SARS-COV-2 pandemic [41, 76]. The 2nd key gene SIRT1 is a key regulator of ACE2 levels [78]. The SIRT1 gene could control viral entry of SARS-CoV and viral replication [79–81]. So, SIRT1 inhibitors may be a valid solution to treat novel coronavirus. The 4th key gene PRKACB is strongly linked with SARS-COV-2 infections [82]. The 5th key gene SMAD4 may lead to successful approaches for SARS-associated coronavirus treatment by targeting the TGF-beta signaling pathway and drive downstream of virus-induced apoptosis [83]. The TGF-beta signaling pathway connected genes may play key roles in SARS-CoV infection [84]. Recent studies also demonstrate that the SMAD4 was identified as target hubs for SARS-CoV [85]. The 6th key gene RIPK-I might be involved in the development of acute respiratory distress syndrome (ARDS) or acute lung injury connected with SARS-CoV-2 infections [86, 87]. The RIPK1-dependent cell death activation might be influenced by viral infections [88]. The RIPK1 associated pathways have potential roles to manage SARS-COV-2 infections [89]. The 7th key gene BIRC3 drives the host response against viral infections [90]. It is involved in the immune, inflammatory response and other potential SARS-COV-2 related molecular pathways [40, 91–94]. The 8th key protein ATM plays a central role in responses to various forms of DNA damage, immunodeficiency, virus integration, virus replication and virus infection [95, 96]. The 9th key gene CCT2 is a potential drug target against SARS-COV-2 infections [93, 97, 98]. The 10th key gene ETS1 is associated with some potential SARS-COV-2 infections related molecular pathways [99, 100]. Thus we observed that our proposed HubGs are also strongly associated with SARS-CoV-2 infections and related indications according to previous researches.

The GO functional and KEGG pathway enrichment analyses of DEGs reflected the significant biological processes (BPs), molecular functions (MFs), cellular components (CCs) and pathways that are highly linked with the COVID-19 infection (**Table 1**). Among the enriched BPs, the apoptotic signaling pathway or regulation of apoptotic process directly leads to stop or slow COVID-19 infection progression [101]. The immune responses is correlated with protective immunity against SARS-CoV-2 [102]. The interleukin-8 production is an important biological function of COVID-19 because interleukin-8 (IL-8) is a potential biomarker to identify different disease severity and prognosis of COVID-19 patients [103]. The leukocyte differentiation may strongly control the SARS-CoV-2 since it is interconnected with respiratory viral infections [104]. The regulation of angiogenesis plays a crucial role to lead the respiratory complications such as SARS-CoV-2 [105]. The regulation of cytokine production plays a crucial role as a potential novel biomarker of COVID-19 infection [106]. The SARS-CoV-2 directly is correlated to and contributes to the regulation of programmed cell death [107]. Hypoxia reduces ACE2 expression and inhibits SARS-CoV-2 entry and replication in lung epithelial cells [108]. The hypoxia is a primary pathophysiologic feature and the main cause of mortality in patients with severe COVID-19 and it accompanies all the stages of the disease [109]. The response of oxidative stress makes an important contribution to pathogenesis of the SARS-CoV-2 and other respiratory viruses [110]. Among the enriched MFs, the transcription factor binding sites present in SARS-CoV-2 are involved in the host interferon response, gene influencing and pathways with biological functions which can play a crucial role in SARS-CoV-2 [111, 112]. The secretory granule cellular components is also a key biomarker to replicate and entry of SARS-CoV-2 infection respectively [113, 114]. Among the enriched KEGG pathways, the chemokine signaling pathway is a novel signaling pathway directly involved in the different stages of SARS-CoV-2 infection and also in regulating SARS-CoV and SARS-CoV-2 infectious disease [115, 116]. Some common pathways of various cancers are identified at the host–virus interface of SARS-CoV-2 for rapid drug repurposing to treat infections [89].

The 6 TFs proteins (FOXC1, GATA2, YY1, FOXL1, TP53 and SRF) were treated as the key transcriptional regulatory factors of HubGs (**see Fig 4C**). Among them, the TF-protein GATA2 is associated with Hematopoietic and immune defects which influenced the SARS-CoV-2 [117]. The TF-protein FOXL1 expression is associated with numerous cancer [118], the TF-protein TP53 associated with human lung cancer [119] and the TF-protein SRF in related prostate cancer [120]. The 5 microRNAs (hsa-mir-92a-3p, hsa-mir-155-5p, hsa-mir-106b-5p, hsa-mir-34a-5p and hsa-mir-19b-3p) were treated as the key post-transcriptional regulatory factors of HubGs (see **Fig 4A and S4 Table).** The miRNA hsa-mir-92a-3p miRNA is connected with cell activation in coronary heart disease (CHD) [121]. The miRNA hsa-mir-155-5p leads to activate B and T cells, and immune function [122]. Recent studies demonstrated that the miRNAs hsa-mir-106b-5p, hsa-mir-34a-5p and hsa-mir-19b-are also the promising therapeutics for SARS-CoV-2 [85, 123]. In this study, we also detected 6 chemicals for both transcriptional and post-transcriptional regulatory factors of HubGs (see **Fig 4C** and **S4 Table).** A recent study reported that valproic acid is a potential therapeutic chemical for the COVID-19 [124]. Cyclosporine is also considered as a treatment for SARS [125]. Copper sulfate also considered as a candidate drug against SARS-CoV-2 [126]. Arsenic trioxide has a pivotal role in acute leukemia and leukemia related with COVID-19 [127]. Thus we observed that the key regulatory factors of our proposed HubGs are also associated with SARS-CoV-2 infections and related indications according to previous researches.

The disease versus HubGs interaction network analysis revealed that the identified HubGs are linked with various types of cancers and some other diseases including diabetes and respiratory diseases **(Fig 5 and S5 Table)** that might increase the suffering level or death rate of SARS-COV-2 infected patients. We observed that the ATM gene is connected with maximum number of diseases including respiratory infections, liver carcinoma, Bronchiectasis, diabetes, cellular immunodeficiency, fever, leukemia, fibrosis in the network compare to other HubGs. The SIRT1 gene is associated with lung Injury, autoimmune diseases, liver cirrhosis, HIV infections, diabetes, heart diseases while the MED17, BIRC3 and ETS1 are respectively linked with 20, 19 and 3 diseases including infiltrate of lung, anemia, liver cirrhosis, fever, reperfusion injury, hypsarrhythmia, especially which could be a severe comorbidities for the SARS-COV-2 patients. The multivariate survival analysis of lung cancer patients based on expressions of HubGs indicates their significant associations. Thus from the above discussions, we observed that our proposed genomic biomarkers (HubGs) of SARS-CoV-1 infections and their regulatory factors (TFs, miRNA and chemicals), GO functions and KEGG pathways are also associated with different diseases including SARS-CoV-2 infections. The involvement of HubGs in various diseases is indicating that the patients suffering from coronavirus infections may have greater morbidity and mortality risk than other diseases. The patients having the diseases associated with HubGs may have a greater chance to be affected with SARS-COV-2 infections and their mortality rate might also be significantly higher than normal patients. The phylogenetic tree analysis (**Fig 7)** also showed that SARS-CoV-1 and SARS-CoV-2 are genetically closely related. Therefore, our proposed host genomic biomarkers guided repurposable drugs might be effective for SARS-COV-2 patients suffering from other comorbidities also.

To explore our proposed genomic biomarkers guided repurposable drugs, we considered proposed HubGs based 11 key proteins (SMAD4, GSK3B, SIRT1, ATM, RIPK1, PRKACB, MED17, CCT2, BIRC3, ETS1 and TXN) and their regulatory 6 TFs proteins (FOXC1, GATA2, YY1, FOXL1, TP53 and SRF) as the drug target receptors and performed their docking simulation with the SARS-CoV-2 3CL protease-guided top ranked FDA approved 90 anti-viral drugs out of 3410. Then we selected top ranked 7 drugs (Rapamycin, Tacrolimus, Torin-2, Redotinib, Ivermectin, Danoprevir, Daclatasvir) as the most probable repurposable candidate-drugs for SARS-CoV-1 infections based on their strong binding affinity scores (kCal/mol) with all the target proteins (see **Fig 8A and 8B**). Then, we validated these 7 candidate-drugs against the already published top ranked 11 target proteins (MX1, IRF7, NFKBIA, STAT1, IL6, TNF, CCL20, CXCL8, VEGFA, CASP3, ICAM1) associated with SARS-CoV-2 infections by molecular docking simulation and found their strongly significant binding affinity scores with our proposed candidate-drugs (see **Fig 8C**). Among the identified candidate-drugs, Rapamycin is the prototypic mTOR (Mammalian Target of Rapamycin) inhibitor drug that can be repurposed at low dosages for the potential treatment of SARS-CoV-2 infections and autoimmune lymphoproliferative syndrome and synthetized for effective therapeutic use although it has wide range information about their activity against the SARS-CoV-2 infections [128–131]. The Rapamysin can also interact with the spike protein of the SARS-CoV-2 and work in mTOR pathway inhibitors [132–135]. Tacrolimus is recommended as a potential drug against SARS-COV-2 infections, considering the host virus interaction and potential antiviral drug targets with repurposed drugs [136–139]. Tacrolimus is needed to activate the cellular immune response, possibly for preventing a cytokine storm in severe SARS-COV-2 and it also showed effective inhibition of viral replication of SARS-CoV [140–144]. In-vitro or in-vivo, tacrolimus (TAC) was used as immunosuppressive drugs to treat autoimmune [145]. The Torin-2 kinase inhibitor is considered as potential therapeutics against SARS-COV-2 which worked in the PI3K-Akt/ mTOR signaling pathway [132, 146, 147]. Additionally, Torin-2 worked in antiviral activities and inhibits virus replication [148, 149]. The danoprevir is an anti-viral naive and experienced promising therapeutic option against SARS-COV-2 infections which was confirmed by clinical trial [150–152]. The Daclatasvir has been initiated in some countries for SARS-COV-2 clinical trials. Recently, Daclatasvir (DCV) as a potential treatment for SARS-COV-2 patients which is reported by clinical trial [153–155]. Two studies reported that the Radotinib can be used for the prevention, alleviation, or treatment of viral respiratory disease. Specifically, the present invention can be used as a useful antiviral agent for the prevention or treatment of disease caused by infection of the SARS-CoV-2 and MERS-CoV [156–158]. A molecular docking simulation study suggested that Ivermectin may be an effective treatment for SARS-Cov2 infections [159]. Another study demonstrated that ivermectin is a safe and effective drug candidate against adult patients with mild SARS-Cov2 infections [160]. Thus the literature review also supported our proposed drugs for the treatment against SARS-CoV-2 infections.

## 5 Conclusion

In this article, we attempted to explore commonly effective supporting drugs for the treatment against different variants of coronavirus infections. Selection of both drug target proteins and agents from a large number of alternatives are equally important in drug discovery by molecular docking. Therefore, firstly, we identified 17 drug target proteins (SMAD4, GSK3B, SIRT1, ATM, RIPK1, PRKACB, MED17, CCT2, BIRC3, ETS1, TXN, FOXC1, GATA2, YY1, FOXL1, TP53 and SRF) from a large number of alternatives by analyzing microarray gene-expression profiles of SARS-CoV-1 infected and control samples based on the integrated statistics and bioinformatics approaches. Then, we identified our proposed target proteins guided top-ranked 7 repurposable drugs (Rapamycin, Tacrolimus, Torin-2, Redotinib, Ivermectin, Danoprevir, Daclatasvir) for the treatment against SARS-CoV-1infections. Then, we validated these 7 candidate-drugs against the state-of-the-arts top-ranked 11 target proteins (MX1, IRF7, NFKBIA, STAT1, IL6, TNF, CCL20, CXCL8, VEGFA, CASP3, ICAM1) that are associated with different variants of SARS-CoV-2 infections by molecular docking simulation and found their significant binding affinity scores. The literature review also supported our proposed drugs for the treatment against SARS-CoV-2 infections. Therefore, our findings might be an effective therapeutic resource for the better treatment against different variants of SARS-CoV-2 infections with comorbidities, since our proposed target proteins are also associated with several comorbidities. In the context of already published target proteins and associated repurposable drugs for the treatment against SARS-CoV-2 infections, so far, no other researchers yet investigated the possible efficacy of their suggested drugs against the already published other different variants of receptor proteins (genomic biomarkers) associated with different variants of SARS-CoV-2 infections. As covid-19 is a new coronavirus disease, there has been little research on exploring globally effective drugs. Therefore, our partially unique type research on coronavirus disease might become more and more important with the availability of exceeding sets of target proteins. However, our proposed drugs should be further evaluated in molecular level wet-lab experiments in prior to clinical studies in the treatment of SARS-CoV-2 infections.

## Supporting information

S1 Table Top ranked 90 anti-viral drugs out of 3410 against SARS-CoV-2 infections proposed by Beck et al. [25].
(DOCX)

S2 Table List of significant DEGs between SARS-CoV-1 and control samples, where 86 DEGs are upregulated and the rest 52 DEGs are downregulated, and bold DEGs indicate as Hub-DEGs (HubGs). (DOCX)

S3 Table The list of Hub-DEGs (HubGs) which are selected from protein-protein interaction (PPI) analysis according to topological measure degree (>10) in STRING database. (DOCX)

S4 Table List of key chemicals, TFs and miRNAs those are interacted with DEGs and HubGs of SARS-CoV-1. (DOCX)

S5 Table List of diseases those are associated with at least one HubGs based on DisGeNET database. (DOCX)

## References

[1] Maskalyk J, Hoey J. SARS update. CMAJ. 2003;168(10):1294–5. 12743077 PMC154192

[2] World Health Organization. Summary of probable SARS cases with onset of illness from 1 November 2002 to 31 July 2003. 2004. Available from: http://www.who.int/csr/sars/country/table2004_04_21/en/index.html.

[3] Peiris JS, Yuen KY, Osterhaus AD, Stöhr K. The severe acute respiratory syndrome. New England Journal of Medicine. 2003;349(25):2431–41. doi: 10.1056/NEJMra032498 14681510

[4] Leung GM, Hedley AJ, Ho LM, Chau P, Wong IO, Thach TQ, et al. The epidemiology of severe acute respiratory syndrome in the 2003 Hong Kong epidemic: an analysis of all 1755 patients. Annals of internal medicine. 2004;141(9):662–73. doi: 10.7326/0003-4819-141-9-200411020-00006 15520422

[5] Fouchier RA, Kuiken T, Schutten M, Van Amerongen G, Van Doornum GJ, Van Den Hoogen BG, et al. Koch’s postulates fulfilled for SARS virus. Nature. 2003;423(6937):240. doi: 10.1038/423240a 12748632 PMC7095368

[6] Fowler RA, Lapinsky SE, Hallett D, Detsky AS, Sibbald WJ, Slutsky AS, et al. Critically ill patients with severe acute respiratory syndrome. Jama. 2003;290(3):367–73. doi: 10.1001/jama.290.3.367 12865378

[7] Lew TW, Kwek TK, Tai D, Earnest A, Loo S, Singh K, et al. Acute respiratory distress syndrome in critically ill patients with severe acute respiratory syndrome. Jama. 2003; 290(3):374–80. doi: 10.1001/jama.290.3.374 12865379

[8] Chen Y, Liu Q, Guo D. Emerging coronaviruses: genome structure, replication, and pathogenesis. Journal of medical virology. 2020; 92(4):418–23. doi: 10.1002/jmv.25681 31967327 PMC7167049

[9] Zhu N, Zhang D, Wang W, Li X, Yang B, Song J, et al. China Novel Coronavirus Investigating and Research Team. A novel coronavirus from patients with pneumonia in China, 2019. N Engl J Med. 2020; 382(8):727–33. doi: 10.1056/NEJMoa2001017 31978945 PMC7092803

[10] Zhou P, Yang XL, Wang XG, Hu B, Zhang L, Zhang W, et al. A pneumonia outbreak associated with a new coronavirus of probable bat origin. nature. 2020;579(7798):270–3. doi: 10.1038/s41586-020-2012-7 32015507 PMC7095418

[11] Wu F, Zhao S, Yu B, Chen YM, Wang W, Song ZG, et al. A new coronavirus associated with human respiratory disease in China. Nature. 2020;579(7798):265–9. doi: 10.1038/s41586-020-2008-3 32015508 PMC7094943

[12] Benvenuto D, Giovanetti M, Ciccozzi A, Spoto S, Angeletti S, Ciccozzi M. The 2019‐new coronavirus epidemic: evidence for virus evolution. Journal of medical virology. 2020;92(4):455–9. doi: 10.1002/jmv.25688 31994738 PMC7166400

[13] Xu J, Zhao S, Teng T, Abdalla AE, Zhu W, Xie L, et al. Systematic comparison of two animal-to-human transmitted human coronaviruses: SARS-CoV-2 and SARS-CoV. Viruses. 2020;12(2):244. doi: 10.3390/v12020244 32098422 PMC7077191

[14] Li W, Moore MJ, Vasilieva N, Sui J, Wong SK, Berne MA, et al. Angiotensin-converting enzyme 2 is a functional receptor for the SARS coronavirus. Nature. 2003;426(6965):450–4. doi: 10.1038/nature02145 14647384 PMC7095016

[15] Yan R, Zhang Y, Li Y, Xia L, Guo Y, Zhou Q. Structural basis for the recognition of SARS-CoV-2 by full-length human ACE2. Science. 2020;367(6485):1444–8. doi: 10.1126/science.abb2762 32132184 PMC7164635

[16] Pathogenicity Chen J. and transmissibility of 2019-nCoV—a quick overview and comparison with other emerging viruses. Microbes and infection. 2020;22(2):69–71. doi: 10.1016/j.micinf.2020.01.004 32032682 PMC7102641

[17] Chen L, Liu W, Zhang Q, Xu K, Ye G, Wu W, et al. RNA based mNGS approach identifies a novel human coronavirus from two individual pneumonia cases in 2019 Wuhan outbreak. Emerging microbes & infections. 2020;9(1):313–9. doi: 10.1080/22221751.2020.1725399 32020836 PMC7033720

[18] Zhu RF, Gao YL, Robert SH, Gao JP, Yang SG, Zhu CT. Systematic review of the registered clinical trials for coronavirus disease 2019 (COVID-19). Journal of translational medicine. 2020;18(1):1–9. doi: 10.1186/s12967-019-02189-8 32631442 PMC7338108

[19] Vaccine Centre, London School of Hygiene and Tropical Medicine. 2021. Available from: https://www.lshtm.ac.uk/newsevents/events/series/vaccine-centre

[20] Rahman MR, Islam T, Shahjaman M, Zaman T, Faruquee HM, Jamal MA, et al. Discovering biomarkers and pathways shared by alzheimer’s disease and ischemic stroke to identify novel therapeutic targets. Medicina. 2019;55(5):191. doi: 10.3390/medicina55050191 31121943 PMC6572146

[21] Rahman MR, Islam T, Turanli B, Zaman T, Faruquee HM, Rahman MM, et al. Network-based approach to identify molecular signatures and therapeutic agents in Alzheimer’s disease. Computational biology and chemistry. 2019;78:431–9. doi: 10.1016/j.compbiolchem.2018.12.011 30606694

[22] Shahjaman M, Rahman M, Islam SM, Mollah M, Haque N. A robust approach for identification of cancer biomarkers and candidate drugs. Medicina. 2019;55(6):269. doi: 10.3390/medicina55060269 31212673 PMC6631768

[23] Moni MA, Islam MB, Rahman MR, Rashed-Al-Mahfuz M, Awal MA, Islam SM, et al. Network-based computational approach to identify delineating common cell pathways influencing type 2 diabetes and diseases of bone and joints. IEEE Access. 2019;8:1486–97. doi: 10.1109/ACCESS.2019.2962091

[24] Islam T, Rahman R, Gov E, Turanli B, Gulfidan G, Haque A, et al. Drug targeting and biomarkers in head and neck cancers: insights from systems biology analyses. Omics: a journal of integrative biology. 2018;22(6):422–36. doi: 10.1089/omi.2018.0048 29927717

[25] Beck BR, Shin B, Choi Y, Park S, Kang K. Predicting commercially available antiviral drugs that may act on the novel coronavirus (SARS-CoV-2) through a drug-target interaction deep learning model. Computational and structural biotechnology journal. 2020;18:784–90. doi: 10.1016/j.csbj.2020.03.025 32280433 PMC7118541

[26] Wang Z, Jiang C, Zhang X, Zhang Y, Ren Y, Gao X. Identification of Key Genes and Pathways in SARS-CoV-2 Infection using Bioinformatics Analysis. 2020. doi: 10.21203/rs.3.rs-72821/v1

[27] Gu H, Yuan G. Identification of potential key genes for SARS-CoV-2 infected human bronchial organoids based on bioinformatics analysis. bioRxiv. 2020. doi: 10.1101/2020.08.18.256735

[28] Nan KS, Karuppanan K, Kumar S. Identification of common key genes and pathways between Covid-19 and lung cancer by using protein-protein interaction network analysis. bioRxiv. 2021. doi: 10.1101/2021.02.16.431364

[29] Gu H, Yuan G. Identification of key genes and pathways in the hPSC-derived lungs infected by the SARS-CoV-2. 2020. doi: 10.21203/rs.3.rs-114578/v1

[30] Sardar R, Satish D, Gupta D. Identification of novel SARS-CoV-2 drug targets by host microRNAs and transcription factors co-regulatory interaction network analysis. Frontiers in Genetics. 2020;11:1105. doi: 10.3389/fgene.2020.571274 33173539 PMC7591747

[31] Gu H, Yuan G. Identification of key genes in SARS-CoV-2 patients on bioinformatics analysis. bioRxiv. 2020. doi: 10.1101/2020.08.09.243444

[32] Xie TA, Han MY, Su XR, Li HH, Chen JC, Guo XG. Identification of Hub genes associated with infection of three lung cell lines by SARS‐CoV‐2 with integrated bioinformatics analysis. Journal of cellular and molecular medicine. 2020;24(20):12225. doi: 10.1111/jcmm.15862 32924263 PMC7579704

[33] Oh JH, Tannenbaum A, Deasy JO. Identification of biological correlates associated with respiratory failure in COVID-19. BMC Medical Genomics. 2020; 13(1):1–6. doi: 10.1186/s12920-019-0646-9 33308225 PMC7729705

[34] Vastrad B, Vastrad C, Tengli A. Identification of potential mRNA panels for severe acute respiratory syndrome coronavirus 2 (COVID-19) diagnosis and treatment using microarray dataset and bioinformatics methods. 3 Biotech. 2020;10(10):1–65. doi: 10.1007/s13205-020-02406-y 33251083 PMC7679428

[35] Prasad K, Khatoon F, Rashid S, Ali N, AlAsmari AF, Ahmed MZ, et al. Targeting hub genes and pathways of innate immune response in COVID-19: a network biology perspective. International journal of biological macromolecules. 2020;163:1–8. doi: 10.1016/j.ijbiomac.2020.06.228 32599245 PMC7319641

[36] Selvaraj G, Kaliamurthi S, Peslherbe GH, Wei DQ. Identifying potential drug targets and candidate drugs for COVID-19: biological networks and structural modeling approaches. F1000Research. 2021;10. doi: 10.12688/f1000research.50850.3 33968364 PMC8080978

[37] Satu MS, Khan MI, Rahman MR, Howlader KC, Roy S, Roy SS, et al. Diseasome and comorbidities complexities of SARS-CoV-2 infection with common malignant diseases. Briefings in Bioinformatics. 2021;22(2):1415–29. doi: 10.1093/bib/bbab003 33539530 PMC7929360

[38] Taz TA, Ahmed K, Paul BK, Kawsar M, Aktar N, Mahmud SH, et al. Network-based identification genetic effect of SARS-CoV-2 infections to Idiopathic pulmonary fibrosis (IPF) patients. Briefings in Bioinformatics. 2021 Mar;22(2):1254–66. doi: 10.1093/bib/bbaa235 33024988 PMC7665362

[39] Moni MA, Quinn JM, Sinmaz N, Summers MA. Gene expression profiling of SARS-CoV-2 infections reveal distinct primary lung cell and systemic immune infection responses that identify pathways relevant in COVID-19 disease. Briefings in bioinformatics. 2021 Mar;22(2):1324–37. doi: 10.1093/bib/bbaa376 33333559 PMC7799202

[40] Islam T, Rahman MR, Aydin B, Beklen H, Arga KY, Shahjaman M. Integrative transcriptomics analysis of lung epithelial cells and identification of repurposable drug candidates for COVID-19. European Journal of Pharmacology. 2020;887:173594. doi: 10.1016/j.ejphar.2020.173594 32971089 PMC7505772

[41] Zhou Y, Hou Y, Shen J, Huang Y, Martin W, Cheng F. Network-based drug repurposing for novel coronavirus 2019-nCoV/SARS-CoV-2. Cell discovery. 2020;6(1):1–8. doi: 10.1038/s41421-020-0153-3 32194980 PMC7073332

[42] Ge C, He Y. In Silico prediction of molecular targets of Astragaloside IV for alleviation of COVID-19 Hyperinflammation by systems network pharmacology and Bioinformatic gene expression analysis. Frontiers in pharmacology. 2020;11:1494. doi: 10.3389/fphar.2020.556984 33041797 PMC7525161

[43] Aishwarya S, Gunasekaran K, Margret AA. Computational gene expression profiling in the exploration of biomarkers, non-coding functional RNAs and drug perturbagens for COVID-19. Journal of Biomolecular Structure and Dynamics. 2020:1–6. doi: 10.1080/07391102.2020.1850360 33228475 PMC7754930

[44] Saxena A, Chaudhary U, Bharadwaj A, Wahi N, Kalli JR, Gupta S, et al. A lung transcriptomic analysis for exploring host response in COVID-19. J Pure Appl Microbiol. 2020;14(suppl 1):1077–81. doi: 10.22207/JPAM.14.SPL1.47

[45] Tao Q, Du J, Li X, Zeng J, Tan B, Xu J, et al. Network pharmacology and molecular docking analysis on molecular targets and mechanisms of Huashi Baidu formula in the treatment of COVID-19. Drug development and industrial pharmacy. 2020;46(8):1345–53. doi: 10.1080/03639045.2020.1788070 32643448 PMC7441778

[46] Zhang N, Zhao YD, Wang XM. CXCL10 an important chemokine associated with cytokine storm in COVID-19 infected patients. Eur Rev Med Pharmacol Sci. 2020;24(13):7497–505. doi: 10.26355/eurrev_202007_21922 32706090

[47] Barrett T, Wilhite SE, Ledoux P, Evangelista C, Kim IF, Tomashevsky M, et al. NCBI GEO: archive for functional genomics data sets—update. Nucleic acids research. 2012;41(D1):D991–5. doi: 10.1093/nar/gks1193 23193258 PMC3531084

[48] Reghunathan R, Jayapal M, Hsu LY, Chng HH, Tai D, Leung BP, et al. Expression profile of immune response genes in patients with severe acute respiratory syndrome. BMC immunology. 2005;6(1):1–1. doi: 10.1186/1471-2172-6-2 15655079 PMC546205

[49] Kim S, Chen J, Cheng T, Gindulyte A, He J, He S, et al. PubChem 2019 update: improved access to chemical data. Nucleic acids research. 2019;47(D1):D1102–9. doi: 10.1093/nar/gky1033 30371825 PMC6324075

[50] Law CW, Chen Y, Shi W, Smyth GK. voom: Precision weights unlock linear model analysis tools for RNA-seq read counts. Genome biology. 2014;15(2):1–7. doi: 10.1186/gb-2014-15-2-r29 24485249 PMC4053721

[51] Zhang B, Zhao S, Neuhaus I. canvasDesigner: a versatile interactive high-resolution scientific multi-panel visualization toolkit. Bioinformatics. 2018 Oct 1;34(19):3419–20. doi: 10.1093/bioinformatics/bty377 29726919

[52] Braun P, Gingras AC. History of protein–protein interactions: From egg‐white to complex networks. Proteomics. 2012;12(10):1478–98. doi: 10.1002/pmic.201100563 22711592

[53] Szklarczyk D, Morris JH, Cook H, Kuhn M, Wyder S, Simonovic M, et al. The STRING database in 2017: quality-controlled protein–protein association networks, made broadly accessible. Nucleic acids research. 2016 Oct 18:gkw937. doi: 10.1093/nar/gkw937 27924014 PMC5210637

[54] Xia J, Gill EE, Hancock RE. NetworkAnalyst for statistical, visual and network-based meta-analysis of gene expression data. Nature protocols. 2015;10(6):823–44. doi: 10.1038/nprot.2015.052 25950236

[55] Subramanian A, Tamayo P, Mootha VK, Mukherjee S, Ebert BL, Gillette MA, et al. Gene set enrichment analysis: a knowledge-based approach for interpreting genome-wide expression profiles. Proceedings of the National Academy of Sciences. 2005;102(43):15545–50. doi: 10.1073/pnas.0506580102 16199517 PMC1239896

[56] Huang DW, Sherman BT, Tan Q, Kir J, Liu D, Bryant D, et al. DAVID Bioinformatics Resources: expanded annotation database and novel algorithms to better extract biology from large gene lists. Nucleic acids research. 2007;35(suppl_2):W169–75. doi: 10.1093/nar/gkm415 17576678 PMC1933169

[57] Chen EY, Tan CM, Kou Y, Duan Q, Wang Z, Meirelles GV, et al. Enrichr: interactive and collaborative HTML5 gene list enrichment analysis tool. BMC bioinformatics. 2013;14(1):1–4. doi: 10.1186/1471-2105-14-128 23586463 PMC3637064

[58] Zhou Y, Zhou B, Pache L, Chang M, Khodabakhshi AH, Tanaseichuk O, et al. Metascape provides a biologist-oriented resource for the analysis of systems-level datasets. Nature communications. 2019;10(1):1–0. doi: 10.1038/s41467-018-07882-8 30944313 PMC6447622

[59] Khan A, Fornes O, Stigliani A, Gheorghe M, Castro-Mondragon JA, Van Der Lee R, et al. JASPAR 2018: update of the open-access database of transcription factor binding profiles and its web framework. Nucleic acids research. 2018;46(D1):D260–6. doi: 10.1093/nar/gkx1126 29140473 PMC5753243

[60] Sethupathy P, Corda B, Hatzigeorgiou AG. TarBase: A comprehensive database of experimentally supported animal microRNA targets. Rna. 2006;12(2):192–7. doi: 10.1261/rna.2239606 16373484 PMC1370898

[61] Hsu SD, Lin FM, Wu WY, Liang C, Huang WC, Chan WL, et al. miRTarBase: a database curates experimentally validated microRNA–target interactions. Nucleic acids research. 2011;39(suppl_1):D163–9. doi: 10.1093/nar/gkq1107 21071411 PMC3013699

[62] Davis AP, Grondin CJ, Johnson RJ, Sciaky D, McMorran R, Wiegers J, et al. The comparative toxicogenomics database: update 2019. Nucleic acids research. 2019 Jan 8;47(D1):D948–54. doi: 10.1093/nar/gky868 30247620 PMC6323936

[63] Piñero J, Ramírez-Anguita JM, Saüch-Pitarch J, Ronzano F, Centeno E, Sanz F, et al. The DisGeNET knowledge platform for disease genomics: 2019 update. Nucleic acids research. 2020 Jan 8;48(D1):D845–55. doi: 10.1093/nar/gkz1021 31680165 PMC7145631

[64] Aguirre-Gamboa R, Gomez-Rueda H, Martínez-Ledesma E, Martínez-Torteya A, Chacolla-Huaringa R, Rodriguez-Barrientos A, et al. SurvExpress: an online biomarker validation tool and database for cancer gene expression data using survival analysis. PloS one. 2013;8(9):e74250. doi: 10.1371/journal.pone.0074250 24066126 PMC3774754

[65] Trott O, Olson AJ. AutoDock Vina: improving the speed and accuracy of docking with a new scoring function, efficient optimization, and multithreading. Journal of computational chemistry. 2010;31(2):455–61. doi: 10.1002/jcc.21334 19499576 PMC3041641

[66] Morris GM, Huey R, Lindstrom W, Sanner MF, Belew RK, Goodsell DS, et al. AutoDock4 and AutoDockTools4: Automated docking with selective receptor flexibility. Journal of computational chemistry. 2009;30(16):2785–91. doi: 10.1002/jcc.21256 19399780 PMC2760638

[67] Pettersen EF, Goddard TD, Huang CC, Couch GS, Greenblatt DM, Meng EC, et al. UCSF Chimera—a visualization system for exploratory research and analysis. Journal of computational chemistry. 2004 Oct;25(13):1605–12. doi: 10.1002/jcc.20084 15264254

[68] Visualizer DS. v4. 0.100. 13345. Accelrys Software Inc. 2005;2013.

[69] Schneidman-Duhovny D, Inbar Y, Nussinov R, Wolfson HJ. PatchDock and SymmDock: servers for rigid and symmetric docking. Nucleic acids research. 2005;33(suppl_2):W363–7. doi: 10.1093/nar/gki481 15980490 PMC1160241

[70] Wishart DS, Feunang YD, Guo AC, Lo EJ, Marcu A, Grant JR, et al. DrugBank 5.0: a major update to the DrugBank database for 2018. Nucleic acids research. 2018;46(D1):D1074–82. doi: 10.1093/nar/gkx1037 29126136 PMC5753335

[71] Tamura K, Stecher G, Kumar S. MEGA11: molecular evolutionary genetics analysis version 11. Molecular biology and evolution. 2021;38(7):3022–7. doi: 10.1093/molbev/msab120 33892491 PMC8233496

[72] Islam M, Sultana R, Hasan M, Alam M, Sikdar B, Kamaruzzaman M. Characterization and biocontrol measures of Pseudomonas syringae pv. syringae associated with citrus blast disease. Vegetos. 2020;33(3):555–69. doi: 10.1007/s42535-020-00138-1

[73] Sahu AK, Sreepadmanabh M, Rai M, Chande A. SARS-CoV-2: phylogenetic origins, pathogenesis, modes of transmission, and the potential role of nanotechnology. Virusdisease. 2021;32(1):1–2. doi: 10.1007/s13337-021-00653-y 33644261 PMC7897733

[74] Berman HM, Battistuz T, Bhat TN, Bluhm WF, Bourne PE, Burkhardt K, et al. The protein data bank. Acta Crystallographica Section D: Biological Crystallography. 2002;58(6):899–907. doi: 10.1107/s0907444902003451 12037327

[75] Waterhouse A, Bertoni M, Bienert S, Studer G, Tauriello G, Gumienny R, et al. SWISS-MODEL: homology modelling of protein structures and complexes. Nucleic acids research. 2018;46(W1):W296–303. doi: 10.1093/nar/gky427 29788355 PMC6030848

[76] Rana AK, Rahmatkar SN, Kumar A, Singh D. Glycogen synthase kinase-3: A putative target to combat severe acute respiratory syndrome coronavirus 2 (SARS-CoV-2) pandemic. Cytokine & growth factor reviews. 2020. doi: 10.1016/j.cytogfr.2020.08.002 32948440 PMC7446622

[77] Wu CH, Yeh SH, Tsay YG, Shieh YH, Kao CL, Chen YS, et al. Glycogen synthase kinase-3 regulates the phosphorylation of severe acute respiratory syndrome coronavirus nucleocapsid protein and viral replication. Journal of Biological Chemistry. 2009;284(8):5229–39. doi: 10.1074/jbc.M805747200 19106108 PMC8011290

[78] Jang I, Kim EN, Lim JH, Kim MY, Ban TH, Yoon HE, et al. Effects of resveratrol on the renin-angiotensin system in the aging kidney. Nutrients. 2018;10(11):1741. doi: 10.3390/nu10111741 30424556 PMC6267480

[79] Devaux CA, Rolain JM, Raoult D. ACE2 receptor polymorphism: Susceptibility to SARS-CoV-2, hypertension, multi-organ failure, and COVID-19 disease outcome. Journal of Microbiology, Immunology and Infection. 2020;53(3):425–35. doi: 10.1016/j.jmii.2020.04.015 32414646 PMC7201239

[80] Lin SC, Ho CT, Chuo WH, Li S, Wang TT, Lin CC. Effective inhibition of MERS-CoV infection by resveratrol. BMC infectious diseases. 2017;17(1):1–0. doi: 10.1186/s12879-016-2122-x 28193191 PMC5307780

[81] Weston S, Matthews KL, Lent R, Vlk A, Haupt R, Kingsbury T, et al. A yeast suppressor screen used to identify mammalian SIRT1 as a proviral factor for Middle East respiratory syndrome coronavirus replication. Journal of virology. 2019 Jul 30;93(16):e00197–19. doi: 10.1128/JVI.00197-19 31142674 PMC6675885

[82] Hemmat N, Derakhshani A, Bannazadeh Baghi H, Silvestris N, Baradaran B, De Summa S. Neutrophils, crucial, or harmful immune cells involved in coronavirus infection: a bioinformatics study. Frontiers in Genetics. 2020;11:641. doi: 10.3389/fgene.2020.00641 32582303 PMC7296827

[83] Zhao X, Nicholls JM, Chen YG. Severe acute respiratory syndrome-associated coronavirus nucleocapsid protein interacts with Smad3 and modulates transforming growth factor-β signaling. Journal of Biological Chemistry. 2008;283(6):3272–80. doi: 10.1074/jbc.M708033200 18055455 PMC8740907

[84] Nakao A, Imamura T, Souchelnytskyi S, Kawabata M, Ishisaki A, Oeda E, et al. TGF‐β receptor‐mediated signalling through Smad2, Smad3 and Smad4. The EMBO journal. 1997;16(17):5353–62. doi: 10.1093/emboj/16.17.5353 9311995 PMC1170167

[85] Yousefi H, Poursheikhani A, Bahmanpour Z, Vatanmakanian M, Taheri M, Mashouri L, et al. SARS-CoV infection crosstalk with human host cell noncoding-RNA machinery: An in-silico approach. Biomedicine & Pharmacotherapy. 2020;130:110548. doi: 10.1016/j.biopha.2020.110548 33475497 PMC7386606

[86] Shashaty MG, Reilly JP, Faust HE, Forker CM, Ittner CA, Zhang PX, et al. Plasma receptor interacting protein kinase-3 levels are associated with acute respiratory distress syndrome in sepsis and trauma: a cohort study. Critical Care. 2019;23(1):1–1. doi: 10.1186/s13054-018-2293-5 31253195 PMC6599265

[87] Siempos II, Ma KC, Imamura M, Baron RM, Fredenburgh LE, Huh JW, et al. RIPK3 mediates pathogenesis of experimental ventilator-induced lung injury. JCI insight. 2018 May 3;3(9). doi: 10.1172/jci.insight.97102 29720570 PMC6012515

[88] Ivanisenko NV, Seyrek K, Kolchanov NA, Ivanisenko VA, Lavrik IN. The role of death domain proteins in host response upon SARS-CoV-2 infection: modulation of programmed cell death and translational applications. Cell death discovery. 2020;6(1):1–0. doi: 10.1038/s41420-020-00331-w 33072409 PMC7547561

[89] Tutuncuoglu B, Cakir M, Batra J, Bouhaddou M, Eckhardt M, Gordon DE, et al. The landscape of human cancer proteins targeted by SARS-CoV-2. Cancer discovery. 2020 Jul 1;10(7):916–21. doi: 10.1158/2159-8290.CD-20-0559 32444466 PMC7357668

[90] Rouka E. Hypothesis: Is there a link between the immune response to Human Herpes Virus type 6Α (HHV-6Α) infection and the interaction network (interactome) of the genes encoding the CTSS, PTX3, CHI3L1, Mx1, CXCL16, BIRC3 and BST2 proteins?. Medical hypotheses. 2018;112:47–50. doi: 10.1016/j.mehy.2018.01.011 29447938

[91] Alsamman AM, Zayed H. The transcriptomic profiling of SARS-CoV-2 compared to SARS, MERS, EBOV, and H1N1. PLoS One. 2020;15(12):e0243270. doi: 10.1371/journal.pone.0243270 33301474 PMC7728291

[92] Elkahloun AG, Saavedra JM. Candesartan could ameliorate the COVID-19 cytokine storm. Biomedicine & Pharmacotherapy. 2020;131:110653. doi: 10.1016/j.biopha.2020.110653 32942152 PMC7439834

[93] Qin S, Xia X, Shi X, Ji X, Ma F, Chen L. Mechanistic insights into SARS-CoV-2 epidemic via revealing the features of SARS-CoV-2 coding proteins and host responses upon its infection. Bioinformatics. 2020;36(21):5133–8. doi: 10.1093/bioinformatics/btaa725 32805023 PMC7558794

[94] Fagone P, Ciurleo R, Lombardo SD, Iacobello C, Palermo CI, Shoenfeld Y, et al. Transcriptional landscape of SARS-CoV-2 infection dismantles pathogenic pathways activated by the virus, proposes unique sex-specific differences and predicts tailored therapeutic strategies. Autoimmunity reviews. 2020;19(7):102571. doi: 10.1016/j.autrev.2020.102571 32376402 PMC7252184

[95] Ariumi Y, Trono D. Ataxia-telangiectasia-mutated (ATM) protein can enhance human immunodeficiency virus type 1 replication by stimulating Rev function. Journal of virology. 2006;80(5):2445–52. doi: 10.1128/JVI.80.5.2445-2452.2006 16474151 PMC1395391

[96] Canet-Pons J, Schubert R, Duecker RP, Schrewe R, Wölke S, Kieslich M, et al. Ataxia telangiectasia alters the ApoB and reelin pathway. neurogenetics. 2018;19(4):237–55. doi: 10.1007/s10048-018-0557-5 30343341

[97] Ramesh P, Veerappapillai S, Karuppasamy R. Gene expression profiling of corona virus microarray datasets to identify crucial targets in COVID-19 patients. Gene Reports. 2021;22:100980. doi: 10.1016/j.genrep.2020.100980 33263093 PMC7691848

[98] Hazra S, Chaudhuri AG, Tiwary BK, Chakrabarti N. Matrix metallopeptidase 9 as a host protein target of chloroquine and melatonin for immunoregulation in COVID-19: A network-based meta-analysis. Life sciences. 2020;257:118096. doi: 10.1016/j.lfs.2020.118096 32679150 PMC7361122

[99] Karakurt HU, Pinar Pİ. Integration of transcriptomic profile of SARS-CoV-2 infected normal human bronchial epi-thelial cells with metabolic and protein-protein interaction networks. Turkish journal of biology. 2020 Jun 21;44(SI-1):168–77. doi: 10.3906/biy-2005-115 32595353 PMC7314513

[100] Kumar N, Mishra B, Mehmood A, Athar M, Mukhtar MS. Integrative network biology framework elucidates molecular mechanisms of SARS-CoV-2 pathogenesis. Iscience. 2020;23(9):101526. doi: 10.1016/j.isci.2020.101526 32895641 PMC7468341

[101] Astuti I. Severe Acute Respiratory Syndrome Coronavirus 2 (SARS-CoV-2): An overview of viral structure and host response. Diabetes & Metabolic Syndrome: Clinical Research & Reviews. 2020;14(4):407–12. doi: 10.1016/j.dsx.2020.04.020 32335367 PMC7165108

[102] Shah VK, Firmal P, Alam A, Ganguly D, Chattopadhyay S. Overview of immune response during SARS-CoV-2 infection: lessons from the past. Frontiers in immunology. 2020;11:1949. doi: 10.3389/fimmu.2020.01949 32849654 PMC7426442

[103] Li L, Li J, Gao M, Fan H, Wang Y, Xu X, et al. Interleukin-8 as a biomarker for disease prognosis of coronavirus disease-2019 patients. Frontiers in immunology. 2021;11:3432. doi: 10.3389/fimmu.2020.602395 33488599 PMC7820901

[104] McClain MT, Park LP, Nicholson B, Veldman T, Zaas AK, Turner R, et al. Longitudinal analysis of leukocyte differentials in peripheral blood of patients with acute respiratory viral infections. Journal of Clinical Virology. 2013;58(4):689–95. doi: 10.1016/j.jcv.2013.09.015 24140015

[105] Caccuri F, Bugatti A, Zani A, De Palma A, Di Silvestre D, Manocha E, et al. SARS-CoV-2 infection remodels the phenotype and promotes angiogenesis of primary human Lung endothelial cells. Microorganisms. 2021;9(7):1438. doi: 10.3390/microorganisms9071438 34361874 PMC8305478

[106] Horspool AM, Kieffer T, Russ BP, DeJong MA, Wolf MA, Karakiozis JM, et al. Interplay of Antibody and Cytokine Production Reveals CXCL13 as a Potential Novel Biomarker of Lethal SARS-CoV-2 Infection. Msphere. 2021;6(1):e01324–20. doi: 10.1128/mSphere.01324-20 33472985 PMC7845617

[107] Koupenova M, Corkrey HA, Vitseva O, Tanriverdi K, Somasundaran M, Liu P, et al. SARS-CoV-2 initiates programmed cell death in platelets. Circulation research. 2021;129(6):631–46. doi: 10.1161/CIRCRESAHA.121.319117 34293929 PMC8409903

[108] Wing PA, Keeley TP, Zhuang X, Lee JY, Prange-Barczynska M, Tsukuda S, et al. Hypoxic and pharmacological activation of HIF inhibits SARS-CoV-2 infection of lung epithelial cells. Cell reports. 2021;35(3):109020. doi: 10.1016/j.celrep.2021.109020 33852916 PMC8020087

[109] Serebrovska ZO, Chong EY, Serebrovska TV, Tumanovska LV, Xi L. Hypoxia, HIF-1α, and COVID-19: from pathogenic factors to potential therapeutic targets. Acta Pharmacologica Sinica. 2020;41(12):1539–46. doi: 10.1038/s41401-020-00554-8 33110240 PMC7588589

[110] Chernyak BV, Popova EN, Prikhodko AS, Grebenchikov OA, Zinovkina LA, Zinovkin RA. COVID-19 and oxidative stress. Biochemistry (Moscow). 2020;85(12):1543–53. doi: 10.1134/S0006297920120068 33705292 PMC7768996

[111] di Bari I, Franzin R, Picerno A, Stasi A, Cimmarusti MT, Di Chiano M, et al. Severe acute respiratory syndrome coronavirus 2 may exploit human transcription factors involved in retinoic acid and interferon-mediated response: a hypothesis supported by an in silico analysis. New Microbes and New Infections. 2021;41:100853. doi: 10.1016/j.nmni.2021.100853 33680474 PMC7912353

[112] Chetta M, Rosati A, Marzullo L, Tarsitano M, Bukvic N. A SARS-CoV-2 host infection model network based on genomic human Transcription Factors (TFs) depletion. Heliyon. 2020;6(10):e05010. doi: 10.1016/j.heliyon.2020.e05010 32984567 PMC7501776

[113] Morris A. Effects of pancreatic SARS-CoV-2 infection identified. Nature Reviews Endocrinology. 2021;17(4):192–. doi: 10.1038/s41574-021-00481-6 33627835 PMC7903214

[114] Su H, Wan C, Wang ZD, Gao Y, Li YC, Tang F, et al. Expression of CD147 and Cyclophilin A in Kidneys of Patients with COVID-19. Clinical Journal of the American Society of Nephrology. 2021;16(4):618–9. doi: 10.2215/CJN.09440620 33268502 PMC8092067

[115] Cheng LC, Kao TJ, Phan NN, Chiao CC, Yen MC, Chen CF, et al. Novel signaling pathways regulate SARS-CoV and SARS-CoV-2 infectious disease. Medicine. 2021;100(7). doi: 10.1097/MD.0000000000024321 33607766 PMC7899890

[116] Khalil BA, Elemam NM, Maghazachi AA. Chemokines and chemokine receptors during COVID-19 infection. Computational and structural biotechnology journal. 2021;19:976–88. doi: 10.1016/j.csbj.2021.01.034 33558827 PMC7859556

[117] Collin M, Dickinson R, Bigley V. Haematopoietic and immune defects associated with GATA2 mutation. British journal of haematology. 2015;169(2):173–87. doi: 10.1111/bjh.13317 25707267 PMC4409096

[118] Chen X, Deng M, Ma LI, Zhou J, Xiao Y, Zhou X, et al. Inhibitory effects of forkhead box L1 gene on osteosarcoma growth through the induction of cell cycle arrest and apoptosis. Oncology reports. 2015;34(1):265–71. doi: 10.3892/or.2015.3969 26062977

[119] Wei S, Wang H, Lu C, Malmut S, Zhang J, Ren S, et al. The activating transcription factor 3 protein suppresses the oncogenic function of mutant p53 proteins. Journal of Biological Chemistry. 2014;289(13):8947–59. doi: 10.1074/jbc.M113.503755 24554706 PMC3979409

[120] Lundon DJ, Boland A, Prencipe M, Hurley G, O’Neill A, Kay E, et al. The prognostic utility of the transcription factor SRF in docetaxel-resistant prostate cancer: in-vitro discovery and in-vivo validation. BMC cancer. 2017;17(1):1–3. doi: 10.1186/s12885-016-3022-6 28249598 PMC5333466

[121] Zhang Y, Cheng J, Chen F, Wu C, Zhang J, Ren X, et al. Circulating endothelial microparticles and miR-92a in acute myocardial infarction. Bioscience Reports. 2017;37(2). doi: 10.1042/BSR20170047 28213360 PMC5469331

[122] Rodriguez A, Vigorito E, Clare S, Warren MV, Couttet P, Soond DR, et al. Requirement of bic/microRNA-155 for normal immune function. Science. 2007;316(5824):608–11. doi: 10.1126/science.1139253 17463290 PMC2610435

[123] Demirci MD, Adan A. Computational analysis of microRNA-mediated interactions in SARS-CoV-2 infection. PeerJ. 2020 Jun 5;8:e9369. doi: 10.7717/peerj.9369 32547891 PMC7278893

[124] Unal G, Turan B, Balcioglu YH. Immunopharmacological management of COVID-19: Potential therapeutic role of valproic acid. Medical Hypotheses. 2020;143:109891. doi: 10.1016/j.mehy.2020.109891 32498007 PMC7255327

[125] Russell CD, Haas J. Cyclosporine has a potential role in the treatment of SARS. Journal of Infection. 2013;67(1):84–5. doi: 10.1016/j.jinf.2013.01.004 23396219 PMC7133676

[126] Abdel-Mottaleb MS, Abdel-Mottaleb Y. In search for effective and safe drugs against SARS-CoV-2: Part II] The role of selected salts and organometallics of copper, zinc, selenium, and iodine food supplements. ChemRxiv: Preprint. 2020. doi: 10.26434/chemrxiv.12234743.v1

[127] Gavillet M, Klappert JC, Spertini O, Blum S. Acute leukemia in the time of COVID-19. Leukemia research. 2020;92:106353. doi: 10.1016/j.leukres.2020.106353 32251934 PMC7138175

[128] Pandey S, Pathak SK, Pandey A, Salunke AA, Chawla J, Sharma A, et al. Ivermectin in COVID-19: What do we know?. Diabetes & Metabolic Syndrome. 2020;14(6):1921–1922. doi: 10.1016/j.dsx.2020.09.027 33032231 PMC7521351

[129] Albert S, Serova M, Dreyer C, Sablin MP, Faivre S, Raymond E. New inhibitors of the mammalian target of rapamycin signaling pathway for cancer. Expert opinion on investigational drugs. 2010;19(8):919–30. doi: 10.1517/13543784.2010.499121 20569080

[130] Husain A, Byrareddy SN. Rapamycin as a potential repurpose drug candidate for the treatment of COVID-19. Chemico-biological interactions. 2020 Oct 6:109282. doi: 10.1016/j.cbi.2020.109282 33031791 PMC7536130

[131] Bride KL, Vincent T, Smith-Whitley K, Lambert MP, Bleesing JJ, Seif AE, et al. Sirolimus is effective in relapsed/refractory autoimmune cytopenias: results of a prospective multi-institutional trial. Blood, The Journal of the American Society of Hematology. 2016;127(1):17–28. doi: 10.1182/blood-2015-07-657981 26504182 PMC4705607

[132] Kalathiya U, Padariya M, Mayordomo M, Lisowska M, Nicholson J, Singh A, et al. Highly conserved homotrimer cavity formed by the SARS-CoV-2 spike glycoprotein: a novel binding site. Journal of clinical medicine. 2020;9(5):1473. doi: 10.3390/jcm9051473 32422996 PMC7290299

[133] Kindrachuk J, Ork B, Mazur S, Holbrook MR, Frieman MB, Traynor D, et al. Antiviral potential of ERK/MAPK and PI3K/AKT/mTOR signaling modulation for Middle East respiratory syndrome coronavirus infection as identified by temporal kinome analysis. Antimicrobial agents and chemotherapy. 2015;59(2):1088–99. doi: 10.1128/AAC.03659-14 25487801 PMC4335870

[134] American Society of Health-System Pharmacists (Ashp). Assessment of Evidence for COVID-19-Related Treatments. 2020. Available: https://www.ahfscdi.com/

[135] Zhavoronkov A. Geroprotective and senoremediative strategies to reduce the comorbidity, infection rates, severity, and lethality in gerophilic and gerolavic infections. Aging (Albany NY). 2020;12(8):6492–6510. doi: 10.18632/aging.102988 32229705 PMC7202545

[136] Gordon DE, Jang GM, Bouhaddou M, Xu J, Obernier K, White KM, et al. A SARS-CoV-2 protein interaction map reveals targets for drug repurposing. Nature. 2020;583(7816):459–68. doi: 10.1038/s41586-020-2286-9 32353859 PMC7431030

[137] García-Serradilla M, Risco C, Pacheco B. Drug repurposing for new, efficient, broad spectrum antivirals. Virus research. 2019;264:22–31. doi: 10.1016/j.virusres.2019.02.011 30794895 PMC7114681

[138] van de Veerdonk FL, Netea MG, van Deuren M, van der Meer JW, de Mast Q, Brüggemann RJ, et al. Kallikrein-kinin blockade in patients with COVID-19 to prevent acute respiratory distress syndrome. Elife. 2020;9:e57555. doi: 10.7554/eLife.57555 32338605 PMC7213974

[139] Tian S, Hu W, Niu L, Liu H, Xu H, Xiao SY. Pulmonary pathology of early-phase 2019 novel coronavirus (COVID-19) pneumonia in two patients with lung cancer. Journal of thoracic oncology. 2020;15(5):700–4. doi: 10.1016/j.jtho.2020.02.010 32114094 PMC7128866

[140] Hage R, Steinack C, Schuurmans MM. Calcineurin inhibitors revisited: A new paradigm for COVID-19?. Brazilian Journal of Infectious Diseases. 2020;24:365–7. doi: 10.1016/j.bjid.2020.06.005 32603679 PMC7320855

[141] Carbajo-Lozoya J, Müller MA, Kallies S, Thiel V, Drosten C, Von Brunn A. Replication of human coronaviruses SARS-CoV, HCoV-NL63 and HCoV-229E is inhibited by the drug FK506. Virus research. 2012;165(1):112–7. doi: 10.1016/j.virusres.2012.02.002 22349148 PMC7114512

[142] Schoot TS, Kerckhoffs AP, Hilbrands LB, Van Marum RJ. Immunosuppressive drugs and COVID-19: a review. Frontiers in pharmacology. 2020;11:1333. doi: 10.3389/fphar.2020.01333 32982743 PMC7485413

[143] Guillen E, Pineiro GJ, Revuelta I, Rodriguez D, Bodro M, Moreno A, et al. Case report of COVID‐19 in a kidney transplant recipient: does immunosuppression alter the clinical presentation?. American Journal of Transplantation. 2020;20(7):1875–8. doi: 10.1111/ajt.15874 32198834 PMC7228209

[144] Poulsen NN, von Brunn A, Hornum M, Blomberg Jensen M. Cyclosporine and COVID‐19: Risk or favorable?. American Journal of Transplantation. 2020;20(11):2975–82. doi: 10.1111/ajt.16250 32777170 PMC7436557

[145] Pfefferle S, Schöpf J, Kögl M, Friedel CC, Müller MA, Carbajo-Lozoya J, et al. The SARS-coronavirus-host interactome: identification of cyclophilins as target for pan-coronavirus inhibitors. PLoS pathogens. 2011 Oct 27;7(10):e1002331. doi: 10.1371/journal.ppat.1002331 22046132 PMC3203193

[146] Ramaiah MJ. mTOR inhibition and p53 activation, microRNAs: The possible therapy against pandemic COVID-19. Gene reports. 2020;20:100765. doi: 10.1016/j.genrep.2020.100765 32835132 PMC7324924

[147] Simioni C, Cani A, Martelli AM, Zauli G, Tabellini G, McCubrey J, et al. Activity of the novel mTOR inhibitor Torin-2 in B-precursor acute lymphoblastic leukemia and its therapeutic potential to prevent Akt reactivation. Oncotarget. 2014;5(20):10034–47. doi: 10.18632/oncotarget.2490 25296981 PMC4259403

[148] Kuss-duerkop SK, Wang J, Mena I, White K. Metreveli g, Sakthivel R, et al: Influenza virus differentially activates mTORc1 and mTORc2 signaling to maximize late stage replication. PLoS Pathog. 2017;13:e1006635. doi: 10.1371/journal.ppat.1006635 28953980 PMC5617226

[149] Garcia G Jr, Sharma A, Ramaiah A, Sen C, Purkayastha A, Kohn DB, et al. Antiviral drug screen identifies DNA-damage response inhibitor as potent blocker of SARS-CoV-2 replication. Cell reports. 2021;35(1):108940. doi: 10.1016/j.celrep.2021.108940 33784499 PMC7969873

[150] Chen H, Zhang Z, Wang L, Huang Z, Gong F, Li X, et al. First clinical study using HCV protease inhibitor danoprevir to treat COVID-19 patients. Medicine. 2020;99(48):e23357. doi: 10.1097/MD.0000000000023357 33235105 PMC7710192

[151] Choi J, Horner KA, Carnevale K. Atazanavir. Treasure Island (FL): StatPearls Publishing; 2021. 31869072

[152] Sekhar T. Virtual Screening based prediction of potential drugs for COVID-19. Combinatorial Chemistry & High Throughput Screening. 2020;23. doi: 10.2174/1386207323666200814132149 32798373

[153] World Hepatitis Alliance press release: Hepatitis C drugs may offer an inexpensive treatment option for COVID-19. Available from: https://www.worldhepatitisalliance.org/latest-news/infohep/3548907/hepatitis-c-drugs-may-offer-inexpensive-treatment-option-covid-19.

[154] Sadeghi A, Ali Asgari A, Norouzi A, Kheiri Z, Anushirvani A, Montazeri M, et al. Sofosbuvir and daclatasvir compared with standard of care in the treatment of patients admitted to hospital with moderate or severe coronavirus infection (COVID-19): a randomized controlled trial. Journal of Antimicrobial Chemotherapy. 2020;75(11):3379–85. doi: 10.1093/jac/dkaa334 32812039 PMC7454592

[155] Sacramento CQ, Fintelman-Rodrigues N, Temerozo JR, Dias SD, Ferreira AC, Mattos M, et al. The in vitro antiviral activity of the anti-hepatitis C virus (HCV) drugs daclatasvir and sofosbuvir against SARS-CoV-2. BioRxiv. 2020. doi: 10.1101/2020.06.15.153411 PMC8083231 33880524

[156] Kim DY PM, Cho DJ, Lee GY, Kim HY, Lee HU, Ahn CA, et al. Use of radotinib for prevention or treatment of viral respiratory disease. 2017. Available from: https://www.lens.org/lens/patent/049-460-108-815-945/

[157] Rabaan AA, Al-Ahmed SH, Sah R, Tiwari R, Yatoo MI, Patel SK, et al. SARS-CoV-2/COVID-19 and advances in developing potential therapeutics and vaccines to counter this emerging pandemic. Annals of Clinical Microbiology and Antimicrobials. 2020;19(1):1–37. doi: 10.1186/s12941-019-0343-8 32878641 PMC7464065

[158] Novak J, Rimac H, Kandagalla S, Pathak P, Naumovich V, Grishina M, et al. Proposition of a new allosteric binding site for potential SARS-CoV-2 3CL protease inhibitors by utilizing molecular dynamics simulations and ensemble docking. Journal of Biomolecular Structure and Dynamics. 2021;1–4. doi: 10.1080/07391102.2021.1927845 34018907 PMC8146203

[159] Lehrer S, Rheinstein PH. Ivermectin docks to the SARS-CoV-2 spike receptor-binding domain attached to ACE2. in vivo. 2020;34(5):3023–6. doi: 10.21873/invivo.12134 32871846 PMC7652439

[160] Ahmed S, Karim MM, Ross AG, Hossain MS, Clemens JD, Sumiya MK, et al. A five-day course of ivermectin for the treatment of COVID-19 may reduce the duration of illness. International Journal of Infectious Diseases. 2021;103:214–6. doi: 10.1016/j.ijid.2020.11.191 33278625 PMC7709596

