# Supplementary material for: Identification of host transcriptome-guided repurposable drugs for SARS-CoV-1 infections and their validation with SARS-CoV-2 infections by using the integrated bioinformatics approaches
Source: PLoS One. 2022 Apr 7;17(4):e0266124. doi: 10.1371/journal.pone.0266124 (PMC8989220; doi:10.1371/journal.pone.0266124)
Supplement: S1 Table — [25]. (DOCX) [file pone.0266124.s001.docx]

**S1 Table**. Top ranked 90 anti-viral drugs out of 3410 against SARS-CoV-2 infections proposed by Beck et.al.[25]

| FDA Approval Drug List | | |
| --- | --- | --- |
| 5-nonyloxytryptamine | everolimus | Saracatinib (AZD0530) |
| Abacavir sulfate | Famciclovir | saracatinib |
| Abacavir | foxy-5 | scopolamine |
| Acetylcholine Chloride | Ganciclovir | Sildenafil Citrate |
| Acyclovir | indinavir | Simeprevir |
| Adefovir Dipivoxil | ivermectin | sirolimus |
| Amprenavir (agenerase) | Leuprolide Acetate | somatostatin |
| Apixaban | lisuride | staurosporine |
| Asunaprevir (BMS-650032) | lopinavir | Tacrolimus (FK506) |
| Atazanavir sulfate (BMS-232632-05) | Methscopolamine | Telaprevir (VX-950) |
| Atazanavir | mupirocin | temsirolimus |
| Atropine | naltrindole | Tenofovir Disoproxil Fumarate |
| avermectin | Nelfinavir Mesylate | tenofovir |
| Bacitracin | nelfinavir | thiostrepton |
| Batimastat (BB-94) | nevirapine | Tigecycline |
| batimastat | Octreotide acetate | Tiotropium Bromide |
| Boceprevir | oligomycin-a | torin-2 |
| Bosutinib (SKI-606) | Oseltamivir acid | trichostatin-a |
| bosutinib | Oseltamivir phosphate | Valaciclovir HCl |
| Cidofovir | Oseltamivir | valaciclovir |
| Cyclosporin A | Otilonium Bromide | Valganciclovir HCl |
| cyclosporin-a | Penciclovir | Zanamivir |
| Cyclosporine | Peramivir Trihydrate | zolmitriptan |
| dacinostat | Peramivir |  |
| Daclatasvir (BMS-790052) | Pimecrolimus |  |
| danoprevir | prostaglandin |  |
| Daptomycin | Radotinib(IY-5511) |  |
| Darunavir | Raltegravir (MK-0518) |  |
| demecarium | raltegravir |  |
| Difloxacin HCl | Rapamycin (Sirolimus) |  |
| dinoprostone | Remdesivir |  |
| efavirenz | ribavirin |  |
| Elvitegravir (GS-9137) | Rifabutin |  |
| elvitegravir | Rilpivirine |  |
| Entecavir Hydrate | Ritonavir |  |
| entecavir | Rupatadine Fumarate |  |
| eprosartan | Saquinavir mesylate |  |
| Etomidate | saquinavir |  |
