# Supplementary material for: Identification of host transcriptome-guided repurposable drugs for SARS-CoV-1 infections and their validation with SARS-CoV-2 infections by using the integrated bioinformatics approaches
Source: PLoS One. 2022 Apr 7;17(4):e0266124. doi: 10.1371/journal.pone.0266124 (PMC8989220; doi:10.1371/journal.pone.0266124)
Supplement: S2 Table — (DOCX) [file pone.0266124.s002.docx]

**S2 Table:** List of **s**ignificant DEGs between SARS-CoV-1 and control samples, where 86 DEGs are upregulated and the rest 52 DEGs are downregulated, and **Bold** DEGs indicate as Hub-DEGs (HubGs)

| Down-regulated (n = 52) | Up-regulated (n = 86) |
| --- | --- |
| CEACAM8, CHI3L1, BPI, DEFA4, AZU1, HP, RNASE3, LCN2,LTF , CRISP3, CAMP, MS4A3, CEACAM6, RAB13, S100P, ARG1, CYP4F3, ANXA3, TCN1, RNASE2, CENPF, FOXO3, HLX, MC3R, TALDO1, SPAG8, CEBPE, MPO, ST6GALNAC2, MGAM, S100A12, ELANE, PGLYRP1, **CCT2**, GINS2, MMP9, KIF20A, SLPI, EPAS1, ITGAM, CXCR1, ADM, SLC4A1, KCNJ15, TST, MSRB1, NFIL3, DACH1, **TXN**, **GSK3B**, PADI4, GSTO1 | OSGEPL1, MS4A1, RASA3, CREBZF, **PRKACB**, **BIRC3**, EML4, TMOD3, TXK, CR2, CLDND1, HIPK3, AP4E1, LIN7C, EXOC5, **MED17**, GNS, SGPP1, **SMAD4**, KRIT1, NMT2, EIF1AX, BACH2, ARNTL, CDK10, CXCR5, ITGA6, **ATM**, PRPS1, MLLT10, CASP8AP2, LPAR6, **ETS1**, ATP5F1C, FLI1, ANKH, RNFT1, CAPRIN1, TNFRSF25, TIA1, TCEA2, EIF2S3, HSF2, STAM2, ADPRM, TRMT11, NELL2, ADH5, MED6, SLC7A6, PPID, AKAP11, PRKCH, NR3C2, PDCD4, CD164, ALDH5A1, PHC1, FANCF, RASA4, CDC14A, **RIPK1**, **SIRT1**, CASP2, PSIP1, IL21R, CLEC2D, KLRG1, EIF4B, AASDHPPT, BCL11B, ITK, RNF6, MAN1C1, PTER, STAT4, NLE1, PPP1R2, COLQ, FKBP5, SLC4A7, IPP, INPP4A, ZNF134, TUBD1, UTRN |
