# Supplementary material for: Identification of host transcriptome-guided repurposable drugs for SARS-CoV-1 infections and their validation with SARS-CoV-2 infections by using the integrated bioinformatics approaches
Source: PLoS One. 2022 Apr 7;17(4):e0266124. doi: 10.1371/journal.pone.0266124 (PMC8989220; doi:10.1371/journal.pone.0266124)
Supplement: S3 Table — (DOCX) [file pone.0266124.s003.docx]

**S3 Table**: The list of Hub-DEGs (HubGs) which are selected from protein-protein interaction (PPI) analysis according to topological measure degree ($>$10) in STRING database

| HubGs Symbols | Degree | Name | Log2(aFC) | adj.*p*-Val |
| --- | --- | --- | --- | --- |
| GSK3B | 29 | glycogen synthase kinase 3 beta | -1.3272 | 0.046337 |
| SIRT1 | 21 | sirtuin 1 | 1.3122 | 0.03801 |
| MED17 | 18 | mediator complex subunit 17 | 1.9898 | 0.01271 |
| PRKACB | 17 | protein kinase cAMP-activated catalytic subunit beta | 2.8853 | 0.002497 |
| SMAD4 | 16 | SMAD family member 4 | 1.6678 | 0.01271 |
| RIPK1 | 16 | receptor interacting serine/threonine kinase 1 | 1.7284 | 0.03801 |
| BIRC3 | 16 | baculoviral IAP repeat containing 3 | 2.1635 | 0.009488 |
| ATM | 15 | ATM serine/threonine kinase | 2.0634 | 0.018208 |
| CCT2 | 12 | chaperonin containing TCP1 subunit 2 | -2.3431 | 0.026338 |
| ETS1 | 12 | "ETS proto-oncogene 1, transcription factor" | 1.9319 | 0.020913 |
| TXN | 11 | Thioredoxin | -1.3014 | 0.045768 |
