# Supplementary material for: Identification of host transcriptome-guided repurposable drugs for SARS-CoV-1 infections and their validation with SARS-CoV-2 infections by using the integrated bioinformatics approaches
Source: PLoS One. 2022 Apr 7;17(4):e0266124. doi: 10.1371/journal.pone.0266124 (PMC8989220; doi:10.1371/journal.pone.0266124)
Supplement: S4 Table — (DOCX) [file pone.0266124.s004.docx]

**S4 Table**: List of key Chemicals, TFs and miRNAs those are interacted with DEGs and HubGs of SARS-CoV-1

(a) Top 5 key miRNAs which are interacted with DEGs and HubGs in TarBase and miRTarBase database

| miRNAs | No. of associated DEGs | Associated HubGs |
| --- | --- | --- |
| hsa-mir-92a-3p | 20 | SMAD4, GSK3B, SIRT1, ATM, |
| hsa-mir-155-5p | 14 | SMAD4, GSK3B, SIRT1, CCT2, ETS1 |
| hsa-mir-106b-5p | 12 | SMAD4, PRKACB, MED17 |
| hsa-mir-34a-5p | 11 | SIRT1, SMAD4, BIRC3 |
| hsa-mir-19b-3p | 10 | SMAD4, PRKACB, ATM |

N.B. HubGs: Hub Genes

1. Top 5 key chemicals that are interacted with DEGs and HubGs in Comparative Toxicogenomics database

| Chemicals | No. Of Associated DEGs | Associated HubGs |
| --- | --- | --- |
| Valproic Acid | 85 | SMAD4, GSK3B, SIRT1, ATM, RIPK1, PRKACB, MED17, CCT2, BIRC3, ETS1 |
| Cyclosporine | 70 | SMAD4, GSK3B, SIRT1, ATM, PRKACB, MED17, BIRC3, ETS1 and TXN |
| Chemichal-1 | 65 | SMAD4, GSK3B, SIRT1, ATM, PRKACB, MED17, BIRC3, ETS1, TXN |
| Copper Sulfate | 54 | TXN, ATM, GSK3B, BIRC3, SIRT1, SMAD4, RIPK1, MED17 |
| arsenic trioxide | 50 | TXN, ATM, GSK3B, BIRC3, SIRT1, ETS1, CCT2, RIPK1 |

N.B. HubGs: Hub Genes; Chemichal-1: (6-(4-(2-piperidin-1-ylethoxy)phenyl))-3-pyridin-4-ylpyrazolo(1,5-a)pyrimidine

(c) Top 6 key TFs proteins that are interacted with DEGs and in JASPAR database

| TFs | No. of associated DEGs | Associated HubGs |
| --- | --- | --- |
| FOXC1 | 91 | SMAD4, SIRT1, ATM, RIPK1, PRKACB, MED17, CCT2, BIRC3 and TXN |
| GATA2 | 65 | GSK3B, PRKACB, MED17, CCT2, ETS1 |
| YY1 | 40 | SMAD4, ATM, CCT2, BIRC3 |
| FOXL1 | 38 | ATM, RIPK1, PRKACB, CCT2, BIRC3, ETS1 |
| TP53 | 30 | GSK3B, ATM, PRKACB, BIRC3, ETS1 |
| SRF | 29 | GSK3B, SIRT1 |

N.B. HubGs : Hub Genes
