# Supplementary material for: Identification of host transcriptome-guided repurposable drugs for SARS-CoV-1 infections and their validation with SARS-CoV-2 infections by using the integrated bioinformatics approaches
Source: PLoS One. 2022 Apr 7;17(4):e0266124. doi: 10.1371/journal.pone.0266124 (PMC8989220; doi:10.1371/journal.pone.0266124)
Supplement: S5 Table — (DOCX) [file pone.0266124.s005.docx]

**S5 Table: List of diseases those are associated with at least one HubGs based on DisGeNET database**

| HubGs | Diseases | HubGs | Diseases | HubGs | Diseases |
| --- | --- | --- | --- | --- | --- |
| ATM | Abnormal spermatogenesis | ATM | Fatigue | ATM | Pancreatic Neoplasm |
| ATM | Adenoid Cystic Carcinoma | ATM | Female hypogonadism syndrome | ATM | Paranasal Sinus Diseases |
| ATM | Alpha 1 foetoprotein abnormal | ATM | Fever | ATM | Peripheral T-Cell Lymphoma |
| ATM | Anorexia | ATM | Fibrosis | ATM | Polycystic Ovary Syndrome |
| ATM | Aplasia/Hypoplasia of the thymus | ATM | Gait abnormality | ATM | Premature canities |
| ATM | Ataxia Telangiectasia | ATM | Hair abnormalities | ATM | Prenatal Exposure Delayed Effects |
| ATM | Autosomal recessive predisposition | ATM | Hematologic Neoplasms | ATM | Prostatic Neoplasms |
| ATM | B-Cell Lymphomas | ATM | Hepatic enzyme increased | ATM | Recurrent bronchitis |
| ATM | Birthmark | ATM | Hodgkin Disease | ATM | Recurrent respiratory infections |
| ATM | Bladder Neoplasm | ATM | Hypogammaglobulinemia | ATM | Schizophrenia |
| ATM | Bone Marrow Diseases | ATM | Immunoglobulin A and IgG2 deficiency | ATM | Sclerocystic Ovaries |
| ATM | Bronchiectasis | ATM | Impaired glucose tolerance | ATM | Seizures |
| ATM | Cafe-au-Lait Spots | ATM | Increased chromosomal breakage | ATM | Short stature |
| ATM | Cardiomyopathy, Dilated | ATM | Leukemia | ATM | Sinusitis |
| ATM | Cataract | ATM | Liver carcinoma | ATM | Skeletal muscle atrophy |
| ATM | Cellular immunodeficiency | ATM | Liver Dysfunction | ATM | Small thymus |
| ATM | Cerebellar Ataxia | ATM | Liver enzymes abnormal | ATM | Splenomegaly |
| ATM | Choreoathetosis | ATM | Liver function abnormal | ATM | Squamous cell carcinoma |
| ATM | Chromosome Breakage | ATM | Lymphadenopathy | ATM | Stomach Neoplasms |
| ATM | Chronic Lymphocytic Leukemia | ATM | Lymphoma | ATM | Strabismus |
| ATM | Congenital hypoplasia of thymus | ATM | Lymphopenia | ATM | Subclinical abnormal liver function |
| ATM | Conjunctival telangiectasis | ATM | Mammary Neoplasms | ATM | Telangiectasia of the skin |
| ATM | Decreased antibody level in blood | ATM | Melanoma | ATM | Thymic hypoplasia or aplasia |
| ATM | Decreased number of CD4+ T cells | ATM | Mucosal telangiectasiae | ATM | Transaminases increased |
| ATM | Decreased to absent deep tendon reflexes | ATM | Muscle degeneration | ATM | Tremor |
| ATM | Defective B cell differentiation | ATM | Muscle Spasticity | ATM | Ventricular Dysfunction |
| ATM | Delayed Puberty | ATM | Myocardial Infarction | ATM | Weight decreased |
| ATM | Diabetes | ATM | Myoclonus | ATM | Epilepsy |
| HubGs | Diseases | HubGs | Diseases | HubGs | Diseases |
| ATM | Dysarthria | MED17 | Seizures | SIRT1 | Nerve Degeneration |
| ATM | Elevated hepatic transaminases | MED17 | Feeding difficulties | SIRT1 | Neurodegenerative Disorders |
| ATM | Neurogenic Muscular Atrophy | MED17 | Global developmental delay | SIRT1 | Non-alcoholic Fatty Liver Disease |
| ATM | Nystagmus | MED17 | Hypsarrhythmia | SIRT1 | Obesity |
| ATM | Dystonic disease | MED17 | Seizures and Brain Atrophy | SIRT1 | Paralysed |
| BIRC3 | Anemia | MED17 | Mental and motor retardation | SIRT1 | Pneumonia |
| BIRC3 | B-Cell Lymphomas | MED17 | Postnatal Progressive | SIRT1 | Prostatic Neoplasms |
| BIRC3 | Bladder Neoplasm | MED17 | Muscle Spasticity | SIRT1 | Psychoses |
| BIRC3 | Constipation | MED17 | Pediatric failure to thrive |  |  |
| BIRC3 | Dyschezia | MED17 | Postnatal microcephaly | SIRT1 | Retinal Diseases |
| BIRC3 | Familial primary gastric lymphoma | MED17 | Progressive disorder | SIRT1 | Schizophrenia |
| BIRC3 | Fatigue | MED17 | Epilepsy | SIRT1 | Shared Paranoid Disorder |
| BIRC3 | Fatty Liver | SIRT1 | Colitis | SIRT1 | Streptococcal pneumonia |
| BIRC3 | Fever | SIRT1 | Acute kidney injury | SIRT1 | Systemic Scleroderma |
| BIRC3 | Hemoglobin low | SIRT1 | Alcohol abuse | SIRT1 | Thrombosis |
| BIRC3 | HIV Infections | SIRT1 | Atherosclerosis | SIRT1 | Wallerian Degeneration |
| BIRC3 | Hyperhidrosis disorder | SIRT1 | Autoimmune Diseases | SIRT1 | Lung Injury |
| BIRC3 | Increased sweating | SIRT1 | Bipolar Disorder | SIRT1 | Mammary Neoplasms |
| BIRC3 | Infiltrate of lung | SIRT1 | Brain Infarction | SIRT1 | Metabolic Syndrome X |
| BIRC3 | Leukemia | SIRT1 | Brain Neoplasms | SIRT1 | Mood Disorders |
| BIRC3 | Liver Cirrhosis | SIRT1 | Ceroid lipofuscinosis |  |  |
| BIRC3 | Mucosa-Associated Lymphoid Tissue Lymphoma | SIRT1 | Autoimmune Encephalomyelitis |  |  |
| BIRC3 | Nausea and vomiting | SIRT1 | Demyelinating Diseases |  |  |
| BIRC3 | Weight decreased | SIRT1 | Diabetes |  |  |
| ETS1 | Celiac Disease | SIRT1 | Fatty Liver |  |  |
| ETS1 | Lupus Erythematosus | SIRT1 | Fibrosis |  |  |
| ETS1 | Reperfusion Injury | SIRT1 | Genomic Instability |  |  |
| MED17 | Autosomal recessive predisposition | SIRT1 | Heart Diseases |  |  |
| MED17 | Clonus | SIRT1 | HIV Infections |  |  |
| MED17 | Cognitive delay | SIRT1 | Hypertrophy |  |  |
| MED17 | Deglutition Disorders | SIRT1 | Impaired glucose tolerance |  |  |
| MED17 | Diffuse cerebral atrophy | SIRT1 | Insulin Resistance |  |  |
| MED17 | Endometriosis | SIRT1 | Liver Cirrhosis |  |  |
| MED17 | Failure to gain weight | SIRT1 | Myocardial Reperfusion Injury |  |  |
